# Reporting and Utilization of Patient‐Reported Outcomes Measures in the Evaluation of Foot Orthoses Treatment: A Systematic Review

**DOI:** 10.1002/jfa2.70148

**Published:** 2026-03-16

**Authors:** Javier Jiménez‐Guillén, Natalia Tovaruela‐Carrión, Marta María Moreno‐Fresco, Alfonso Martínez‐Nova, Pedro V. Munuera‐Martínez

**Affiliations:** ^1^ Department of Podiatry, Faculty of Nursing, Physiotherapy and Podiatry University of Seville Seville Spain; ^2^ Nursing Department Podiatric Clinic of University of Extremadura CPUEX Plasencia Spain

**Keywords:** foot orthoses, lower extremity, patient‐reported outcome measures, systematic review

## Abstract

**Purpose:**

Currently, there is no patient‐reported outcome measure (PROM) specifically for evaluating foot orthoses treatments. This led us to ask the following question: What is the quality of the validated foot and ankle PROMs used to evaluate foot orthosis interventions? To do so, we analyzed the psychometric properties of these PROMs and identified the most widely used and those with the best measurement properties.

**Methods:**

Two literature searches were conducted in PubMed, Embase, Scopus, Web of Science, and the Cochrane Library. The methodological quality of the articles was assessed using the updated COSMIN checklist. Psychometric evidence for the properties investigated in the articles was assessed using the updated COSMIN criteria for good psychometric properties. Ratings of methodological quality and psychometric evidence were synthesized using the method of Schellingerhout et al. (2012).

**Results:**

A total of 205 articles were included, identifying 11 validated PROMs for foot and ankle used in the evaluation of foot orthoses. The Foot Function Index (FFI) and the Foot Health Status Questionnaire (FHSQ) were the most commonly used, whereas the Victorian Institute of Sport Assessment‐Achilles tendon (VISA‐A) questionnaire demonstrated the best measurement properties.

**Conclusion:**

The VISA‐A, the Foot and Ankle Outcome Score (FAOS), and the Revised Foot Function Index (FFI‐R) appear to be useful PROMs for evaluating foot orthoses treatments; however, more evidence is needed to make more robust and reliable statements.

## 1 Introduction

Foot orthoses are widely prescribed in podiatry and rehabilitation to provide mechanical support at the foot and ankle. They are used to prevent or slow the progression of deformities, alleviate pain and inflammation, redistribute plantar pressures in both static and dynamic conditions, and ultimately enhance foot function [1, 2, 3]. Given their frequent use across a broad range of musculoskeletal and systemic conditions, it is essential to evaluate their effectiveness not only through clinical or biomechanical outcomes but also from the patient's perspective.

Patient‐reported outcome measures (PROMs) are standardized instruments, usually questionnaires, designed to capture a patient's perception of their health status, symptoms, or functional limitations without interpretation by clinicians or third parties [4, 5, 6]. In foot and ankle research, several PROMs have been validated and are frequently employed to assess clinical outcomes. However, none of these instruments were specifically developed to evaluate the effects of foot orthoses. This lack of a tailored PROM represents a gap in the assessment of orthotic interventions, where capturing patient‐reported benefit is critical. The use of PROMs with poor or insufficiently validated measurement properties may lead to inaccurate or misleading conclusions regarding treatment effectiveness, potentially obscuring true patient benefit or harm. This can ultimately affect clinical decision‐making and compromise the comparability of findings across studies.

Against this background, the present review pursued the following objectives: (1) to systematically analyze the psychometric properties of validated PROMs for the foot and ankle that have been used in studies assessing foot orthoses; (2) to identify which of these PROMs are most frequently employed in this context; and (3) to determine which PROMs demonstrate the strongest measurement properties for evaluating orthotic interventions.

## 2 Methods

### 2.1 Protocol and Registration

This review was conducted in accordance with the PRISMA‐COSMIN checklist [7] for systematic reviews of outcome measurement instruments. The protocol was prospectively registered in the International Prospective Register of Systematic Reviews (PROSPERO; registration number CRD42025630582) and is accessible at https://www.crd.york.ac.uk/PROSPERO/view/CRD42025630582.

### 2.2 Research Question

The research question guiding this review was *What is the quality of validated PROMs for the foot and ankle that have been used to evaluate foot orthosis interventions?* The question was structured as follows:

*Participants*: Individuals of any age and sex with a lower limb condition.

*Intervention*: Foot orthoses (insoles).

*Comparison*: Placebo devices or any other treatments prescribed for lower limb conditions.

*Outcomes*: Pain reduction, improved function, enhanced comfort, and better quality of life. These outcomes were proposed to ensure consistency with the expected effects of the intervention. Nevertheless, the focus of this review was not on the clinical outcomes of foot orthoses themselves, but on the PROMs used to measure them in study participants.

*Study design*: Primarily randomized controlled trials were included, as well as clinical trials without a control group but with at least two intervention arms (e.g., a foot orthosis group compared with an alternative intervention).

### 2.3 Search Strategy

Two separate searches were carried out in the following databases: PubMed, Embase, Scopus, Web of Science, and the Cochrane Library (Appendix 1: most recent database searches).

The first search aimed to identify studies addressing the research question, specifically to determine which validated foot‐ and ankle‐related PROMs have been used to evaluate foot orthosis interventions in individuals with lower limb conditions. This search strategy, detailed in Supporting Information S1, was developed using DeCS/MeSH (Health Sciences Descriptors) terms relevant to the topic.

Once the eligible studies had been selected and the PROMs of interest identified, a second search was conducted to locate studies that examined the psychometric properties of those PROMs. For this second search (Supporting Information S1), the names of the identified PROMs were combined with the search filter proposed by Terwee et al. (2009) [8], which is specifically designed to identify studies on measurement properties.

### 2.4 Eligibility Criteria

For the first search, inclusion criteria were defined according to the sections of the research question, with no restrictions on the publication year or language. Only validated foot‐ and ankle‐specific PROMs were considered eligible at this stage. Instruments that were not validated, validated for other body regions, or designed to assess general health status or overall quality of life were excluded. For the second search, inclusion criteria differed: all articles that originally validated the identified foot‐ and ankle‐related questionnaires used to evaluate foot orthoses were eligible, regardless of publication year, language, or study design.

After removing duplicates, references from both searches were screened independently by two reviewers at the title, abstract, and full‐text levels. The reviewers subsequently compared their assessments and resolved any uncertainties through discussion. In cases of disagreement, a third reviewer adjudicated to reach consensus.

### 2.5 Assessment of Methodological Quality

Articles retrieved from the first search were not assessed for methodological quality, as they were used solely to identify validated foot‐ and ankle‐related PROMs applied in studies of foot orthoses. In contrast, articles from the second search were evaluated for methodological quality, since their information directly influenced the results of this review. Quality appraisal was performed using the updated COSMIN risk of bias checklist (version 3.0) [5], a tool specifically developed to evaluate studies on PROMs and their measurement properties.

The methodological quality of studies assessing the following measurement properties was examined: content validity, structural validity, internal consistency, reliability, measurement error, criterion validity, hypothesis testing for construct validity (including convergent and discriminant validity), and responsiveness. COSMIN defines nine key measurement properties of PROMs [5]; in this review, eight were evaluated. Cross‐cultural validity/measurement invariance was not assessed because the included articles primarily involved validation of PROMs into other languages, without considering or testing measurement invariance across distinct cultural or linguistic subgroups.

The COSMIN checklist provides four possible ratings for methodological quality: *very good*, *adequate*, *doubtful*, or *inadequate*. Final scores for each measurement property were assigned according to the “worst score counts” principle, whereby the overall rating for a property is determined by the lowest score on any individual item. For example, if a property was rated “very good” on one item but “inadequate” on another, the overall methodological quality for that property was “inadequate” [5].

All ratings were assigned independently by two reviewers. Disagreements were resolved through discussion, and when consensus could not be reached, a third reviewer acted as arbitrator.

The most frequent methodological shortcomings that affected quality ratings are summarized below:

- *Content validity*: Most studies only evaluated comprehensibility, with little or no consideration of relevance or comprehensiveness. Limitations included unclear assessment methods, unrepresentative samples, and uncertainty about whether all items were evaluated.
- *Structural validity*: Use of inadequate sample sizes relative to the total number of PROM items.
- *Internal consistency*: Lack of prior evidence for unidimensionality and failure to report Cronbach's α values.
- *Reliability*: Inadequate test–retest stability, inappropriate time intervals between assessments, or inconsistent measurement conditions.
- *Measurement error*: In addition to the issues noted under reliability, many studies did not report indices such as the minimal detectable change (MDC), limits of agreement (LoA), or minimal important change (MIC).
- *Criterion validity*: Use of comparators that did not qualify as a gold standard.
- *Construct validity (hypothesis testing)*: Lack of predefined hypotheses or expected directions of results for convergent and discriminant validity.
- *Responsiveness*: Absence of predefined hypotheses regarding expected changes following interventions.

### 2.6 Assessment of Psychometric Evidence

Each article from the second search was also evaluated for the strength of its psychometric evidence, that is, whether the results supported the adequacy of the analyzed measurement properties.

The updated COSMIN criteria for good measurement properties (version 2.0) [5] were applied. According to these criteria, each property was rated as positive (+), indeterminate (?), or negative (−). Table 1 summarizes these criteria in abbreviated form [5].

**TABLE 1:** COSMIN criteria for good measurement properties (version 2.0) [5].

Psychometric property	+ Rating	? Rating	− Rating
Content validity	Items are relevant, appropriate, and comprehensive for the construct, population, and context. Adequate language and comprehensibility; no key concepts missing.	Insufficient information.	Items are not relevant, inappropriate, or incomprehensible; key concepts missing.
Structural validity	Established criteria for classical test theory (CTT) or item response theory (IRT)/Rasch models are met.	Insufficient information.	Established criteria for a positive rating are not met.
Internal consistency	At least low evidence for sufficient unidimensionality and Cronbach's α ≥ 0.70.	Insufficient information or unidimensionality not confirmed.	Low evidence for sufficient unidimensionality or Cronbach's α < 0.70.
Reliability	ICC or (weighted) kappa ≥ 0.70, or Pearson/Spearman correlation ≥ 0.70.	Insufficient information.	ICC, kappa, or correlation < 0.70.
Measurement error	SDC or LoA < MIC.	MIC not defined or insufficient information.	SDC or LoA > MIC.
Criterion validity	Correlation with gold standard or AUC ≥ 0.70.	Insufficient information.	Correlation with gold standard or AUC < 0.70.
Hypothesis testing for construct validity	≥ 75% of results are in accordance with predefined hypotheses.	No relevant results found.	≥ 75% of results deviate from predefined hypotheses.
Responsiveness	≥ 75% of results are in accordance with predefined hypotheses or AUC ≥ 0.70.	No relevant results found.	≥ 75% of results deviate from predefined hypotheses or AUC < 0.70.

Abbreviations: AUC = Area under the receiver operating characteristic curve, CTT = Classical test theory theory, ICC = Intraclass correlation coefficient, IRT/Rasch = Item response theory, LoA = Limits of agreement, MIC = Minimal important change change, SDC = Smallest detectable change change, α = alfa.

As with methodological quality, psychometric evidence ratings were assigned independently by two reviewers. Discrepancies were resolved through discussion, with arbitration by a third reviewer when necessary.

### 2.7 Synthesis Method

The ratings for methodological quality and psychometric evidence of the included studies were synthesized using the approach proposed by Schellingerhout et al. (2012) [9], which has been applied in previous systematic reviews of PROMs [4, 10].This method integrates the consistency of psychometric findings with the methodological quality of the included studies and the levels of evidence recommended by the Cochrane Back Review Group [11].

According to this method, overall results are classified as positive (+), indeterminate (?), negative (−), or no evidence (0), with the level of evidence ranging from *unknown* to *strong* (Table 2). When an equal number of positive and negative ratings are found, the result is classified as conflicting (+/−) [9].

**TABLE 2:** Levels of evidence for the overall quality of the psychometric properties [9].

Level	Rating	Criteria
Strong	+ + + o − − −	Consistent findings in multiples studies of good methodological quality or in one study of excellent methodological quality
Moderate	+ + o − −	Consistent findings in multiples studies of fair methodological quality or in one study of good methodological quality
Limited	+ o −	One study of fair methodological quality
Conflicting	+/−	Conflicting findings
Unknown	?	Only studies of poor methodological quality

Although the COSMIN guideline proposes a structured approach for evidence synthesis, the method by Schellingerhout et al. [9] was selected because it allows a transparent integration of methodological quality, consistency of findings, and levels of evidence across studies, particularly when psychometric evidence is heterogeneous. This approach has been widely applied and accepted in previous systematic reviews of PROMs and was considered appropriate given the variability in study designs and reporting observed in the included literature.

## 3 Results

### 3.1 Study Selection

**FIGURE 1:**
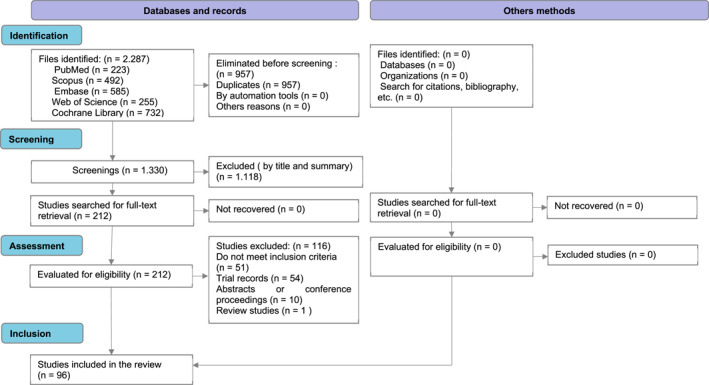
PRISMA‐COSMIN flow diagram of the article selection from the first bibliographic search. *Template from*: Elsman et al. [7].

**FIGURE 2:**
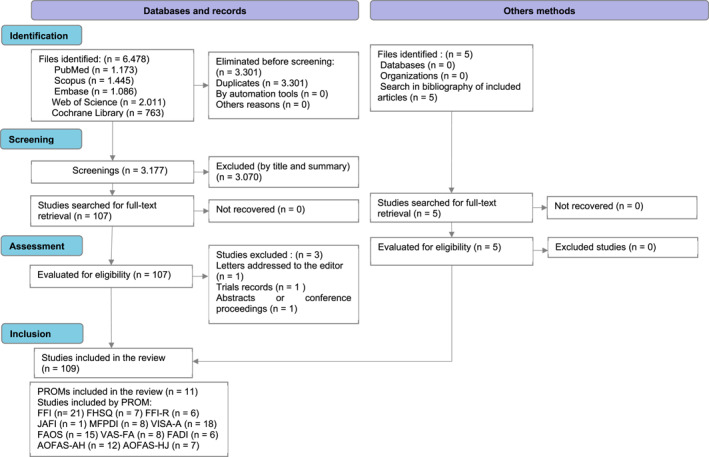
PRISMA‐COSMIN flow diagram of the article selection from the second bibliographic search. *Template from*: Elsman et al. [7].

The selection process for the articles retrieved in the first and second literature searches is presented in Figures 1 and 2, respectively. The flow diagrams were prepared in accordance with the PRISMA‐COSMIN recommendations for systematic reviews of outcome measurement instruments [7].

In addition, supplementary sources were consulted to identify relevant studies for the second search. Specifically, the reference lists of the included articles were screened, yielding five additional studies: one describing the cross‐cultural adaptation of a PROM into another language [12] and four reporting the original development of a PROM [13, 14, 15, 16].

### 3.2 Identified PROMs

Analysis of the studies from the first search yielded a total of 82 PROMs. However, only 11 of these (Table 3) were validated for the foot and ankle and had been used in studies of foot orthosis interventions. The remaining instruments were either not validated, validated for other body regions (e.g., knee, hip), or designed to measure broader constructs such as well‐being or general quality of life rather than being specific to the foot and ankle. Among the 11 PROMs identified, the FFI [17] and the FHSQ [18] were the most frequently used to evaluate foot orthosis treatments (see Supporting Information S1 for the complete list of 82 PROMs and the corresponding references). Of the 11 PROMs identified, only the Juvenile Arthritis Foot Disability Index (JAFI) was specifically developed and validated for pediatric populations with juvenile idiopathic arthritis. The remaining 10 instruments were originally developed and validated for adult populations.

**TABLE 3:** Details of the 11 PROMs identified to evaluate foot orthoses.

PROM (original reference)	Year of publication	No of ítems and domains	Domains	Response options
The foot function index (FFI) [17]	1991	23 items in 3 domains	Pain, disability, and activity limitations	VAS^a^
The foot health status questionnaire (FHSQ) [18]	1985	13 items in 4 domains	Pain, function, footwear, and general foot health	Five point Likert scale
The revised foot function index (FFI‐R) [19]	2006	34 or 68 items in 4 domains	Pain, stiffness, difficulty, activity restriction, and socio‐emotional problems	Six points Likert scale
The visual analog scale‐foot and ankle (VAS‐FA) [15]	2006	20 items in 3 domains	Pain, function, and other discomforts	VAS
The juvenile arthritis disability index for foot and ankle (JAFI) [20]	2004	27 items in 3 domains	Physical disability, activity limitation, and activity restriction	Five points Likert scale
The Manchester foot pain and disability index (MFPDI) [14]	2000	19 items in 3 domains	Pain intensity, functional limitation and personal appearance	Five points Likert scale
The Victorian Institute of Sport Assessment‐Achillestendon Questionnaire (VISA‐A) [21]	2001	8 items in 3 domains	Pain, function in daily life, and sport activity	Item dependent
The Foot and Ankle Outcome Score(FAOS) [22]	2001	42 items in 5 domains	Pain, other symptoms, daily life activities, sports and recreational functions and quality of life related to the foot and ankle	Five points Likert scale
The Foot and Ankle Disability Index (FADI) [16]	1999	26 items in 2 domains	Daily life activities and pain	Five points Likert scale
The American Orthopedic Foot and Ankle Society Clinical Rating Scale‐ankle/hind foot (AOFAS‐AH) [13]	1994	9 items in 3 domains	Pain, function, and alignment	Item‐dependent
The American Orthopedic Foot and Ankle Society clinical rating scale‐hallux metatarsophalangeal‐ interphalangeal joints (AOFAS‐HJ) [13]	1994	8 items in 3 domains	Pain, function, and alignment	Item‐dependent

^a^ VAS = Visual Analog Scale.

### 3.3 Methodological Quality and Psychometric Evidence

The ratings for methodological quality and psychometric evidence are provided in Supporting Information S1.

For content validity, scores referred exclusively to comprehensibility. Most studies were rated as having *doubtful* or *inadequate* methodological quality, and the level of evidence was generally classified as *unknown* or *indeterminate* due to insufficient reporting.

Although there was considerable variability across properties, some—such as internal consistency, reliability, and convergent validity—frequently received positive ratings for psychometric evidence, with several studies also judged to be of *adequate* or *very good* methodological quality. In contrast, structural validity showed the highest number of negative ratings for psychometric evidence.

Other properties, including measurement error, criterion validity, discriminant validity, and responsiveness, were rarely assessed. This highlights a clear lack of evidence, with the corresponding studies generally rated as *inadequate* in methodological quality.

### 3.4 Overall Scores for Psychometric Properties

Using the synthesis method proposed by Schellingerhout et al. (2012) [9], the ratings for methodological quality and psychometric evidence were combined to generate overall scores for each psychometric property of every included PROM (Table 4).

**TABLE 4:** Overall scores for each pyschometric property for each PROM included.

PROM	Content validity	Structural validity	Internal consistency	Reliability	Measurement error	Criterion validity	Construct validity		Responsiveness
							C	D	
FFI	++	++	+++	+++	?	?	+++	0	?
FHSQ	+	+++	+++	+++	?	?	+++	?	0
FFI‐R	+	−	+++	+++	++	0	++	?	+++
JAFI	?	0	?	++	0	0	+++	?	0
MFPDI	+	+++	+++	+++	?	?	+++	?	− − −
VISA‐A	++	+++	+++	+++	?	?	+++	+++	+
FAOS	++	++	+++	+++	?	0	+++	?	+
VAS‐FA	+/−	− −	+++	+++	++	?	++	?	?
FADI	+	?	?	+++	?	?	+	?	?
AOFAS‐AH	+	− −	?	+++	+	0	+++	?	+/−
AOFAS‐HJ	++	0	?	+++	?	0	+++	0	?

*Note:* +++ strong evidence; ++ moderate evidence; + limited evidence; +/− conflicting evidence; ? indeterminate evidence; 0 no evidence.

Abbreviations: C = Convergent validity, D = Discriminative validity.

The VISA‐A questionnaire [21] demonstrated the strongest psychometric performance, with positive ratings for seven properties (content validity, structural validity, internal consistency, reliability, convergent validity, discriminant validity, and responsiveness) and unknown evidence for two properties (measurement error and criterion validity).

Two PROMs showed positive evidence for six properties: the FAOS [22] and the FFI‐R [19]. The FAOS demonstrated positive evidence for content validity, structural validity, internal consistency, reliability, convergent validity, and responsiveness. In turn, the FFI‐R was rated positively for content validity, internal consistency, reliability, measurement error, convergent validity, and responsiveness.

A second group of PROMs—including the FFI [17], the FHSQ [18] and the MFPDI [14]—showed positive evidence for five properties each. This was followed by two PROMs with positive evidence for four properties (the VAS‐FA [15] and the AOFAS‐AH [13]) and two with positive evidence for three properties (the FADI [16] and the AOFAS‐HJ [13]).

The JAFI [20] obtained the lowest ratings, with positive evidence limited to two properties (reliability and convergent validity), whereas the remaining properties were classified as unknown or showed no evidence.

## 4 Discusion

The primary objective of this review was to analyze the psychometric properties of validated foot‐ and ankle‐specific PROMs used to evaluate foot orthosis interventions. Overall, the findings suggest that most of these PROMs demonstrate acceptable measurement properties. One secondary objective was to identify which PROMs were most frequently used in studies of foot orthoses. The FFI [17] and the FHSQ [18] emerged as the most widely validated and commonly applied instruments in this context.

The other secondary objective was to determine which PROMs exhibited the strongest psychometric properties when used in studies of foot orthoses. The VISA‐A questionnaire, designed to assess the severity of Achilles tendinopathy [21], showed the best overall performance, with positive evidence for seven measurement properties. However, despite this strong psychometric performance, its applicability for the general evaluation of foot orthoses is limited, as it is condition‐specific and was used in only one of the studies identified in the first search [23]. In contrast, the FAOS [22] and the FFI‐R [19] also demonstrated strong psychometric performance, with positive evidence for six measurement properties each, while offering broader applicability across a wide range of foot and ankle conditions. When both psychometric quality and clinical applicability are considered, these characteristics make the FAOS and the FFI‐R the most suitable instruments for the general assessment of foot orthosis interventions.

### 4.1 Analysis for Each Psychometric Property

A more detailed analysis of which PROMs achieved the highest ratings for each psychometric property is presented below:

- *Content validity (comprehensibility)*: Most PROMs demonstrated positive, though limited to moderate, evidence (+/++). The only questionnaire with conflicting evidence (+/−) was the VAS‐FA [15], based on findings from two studies [24, 25]. Overall, however, the remaining PROMs appeared to provide acceptable comprehensibility for both patients and clinicians. Nevertheless, it should be noted that, in most studies, content validity was assessed exclusively in terms of comprehensibility, whereas other key aspects such as relevance and comprehensiveness were rarely evaluated. According to COSMIN recommendations, content validity is considered the most important measurement property and insufficient evaluation of relevance and comprehensiveness may limit confidence in whether PROM items fully capture all relevant aspects of the construct from the patient's perspective. This represents a critical methodological limitation of the existing literature and may affect the interpretability and applicability of PROM scores when evaluating the effects of foot orthosis interventions.
- *Structural validity*: Several PROMs demonstrated strong positive evidence (+++), including the MFPDI [26], the FHSQ [27, 28], and the VISA‐A [29, 30]. Consequently, the MFPDI [14], FHSQ [18], and VISA‐A [21] were identified as the instruments with the strongest support for the unidimensionality of their underlying constructs.
- *Internal consistency*: Seven questionnaires—the FFI [17], FHSQ [18], FFI‐R [19], MFPDI [14], VISA‐A [21], FAOS [22], and VAS‐FA [15]—demonstrated strong positive evidence (+++). Among these, the FFI and VISA‐A were supported by the largest number of high‐quality studies [17, 29, 30, 31, 32, 33, 34, 35, 36, 37]. These findings suggest that these seven PROMs show very good internal consistency, indicating strong correlations among their items when measuring the construct of interest.
- *Reliability*: All PROMs showed strong positive evidence (+++), with the exception of the JAFI [20], which demonstrated moderate positive evidence (++). Overall, the identified PROMs appear to be reliable instruments, capable of consistently detecting true differences between patients.
- *Measurement error*: Most PROMs provided only indeterminate evidence (?), with the exception of the FFI‐R [19] and VAS‐FA [15], which showed moderate positive evidence (++) in two studies [25, 38]. Limited positive evidence (+) was also reported for the AOFAS‐AH [39]. Although these instruments appear to have some capacity to assess measurement error, caution is warranted given the overall paucity of evidence for this property.
- *Criterion validity*: This was the psychometric property with the weakest evidence. Five PROMs—theFFI‐R [19], JAFI [20], FAOS [22], AOFAS‐AH [13], and AOFAS‐HJ [13]—showed no available evidence for criterion validity, whereas the remaining PROMs were rated as indeterminate (?). These findings suggest that the identified PROMs lack adequate criterion validity, largely due to the absence of established gold standards for comparison.
- *Hypothesis testing for construct validity*: A distinction must be made between convergent and discriminant validity. For convergent validity, all PROMs demonstrated strong to moderate positive evidence (+++/++). The only exception was the FADI [16], which showed limited positive evidence (+) in the study by Akulaev et al. (2023) [40]. Overall, the PROMs identified appear to have a strong ability to yield results consistent with those of instruments measuring similar constructs.
  For discriminant validity, the findings were markedly different. Only the VISA‐A [21] demonstrated positive evidence, with strong support (+++) reported by Chang et al. (2021) [30], De Mesquita et al. (2018) [41], and Hernández‐Sánchez et al. (2108) [35]. In contrast, the remaining PROMs showed indeterminate (?) or no evidence (0). These results suggest that the VISA‐A has adequate discriminant validity, demonstrating the ability to distinguish between patient groups with different characteristics.
- *Responsiveness*: This property showed the greatest variability in ratings. Only three PROMs—the FFI‐R [19], VISA‐A [21], and FAOS [22]—received positive ratings. Among these, the FFI‐R was the only instrument with strong positive evidence (+++), as reported by Mork et al. (2022) [38]. Although further evidence is required, these three PROMs—particularly the FFI‐R—appear to have adequate responsiveness, reflecting their capacity to detect changes in scores for a construct over time.

The JAFI [20] consistently showed the lowest ratings, with little or no evidence available for most measurement properties. Only the original development study by André et al. (2004) and subsequent cross‐cultural adaptation studies were identified, with no additional validation studies assessing other measurement properties. This limited body of evidence restricts robust conclusions regarding the overall psychometric performance and clinical usefulness of the JAFI.

### 4.2 Strengths and Limitations of the Review

This systematic review has several strengths. First, to our knowledge, it is the first review to evaluate both the methodological quality and the psychometric evidence of validated foot‐ and ankle‐specific PROMs used in studies of foot orthoses. Second, the review applied updated COSMIN criteria to rate methodological quality and psychometric evidence, ensuring a rigorous evaluation framework. Third, two independent searches were performed across five major databases, using health sciences descriptors and established search strategies designed to identify studies on measurement instruments [8]. An additional strength is that study selection, quality appraisal, and evidence synthesis were performed independently by multiple reviewers, which reduced the risk of bias in the evaluation process.

Nevertheless, this review also has limitations. In many cases, the included studies provided incomplete reporting, which made the evaluation more difficult and may have influenced the results. In addition, the evidence synthesis method recommended by COSMIN was not used. Instead, we applied the approach proposed by Schellingerhout et al. (2012) [9], which has been used in other systematic reviews of PROMs [4, 10] and has been accepted in peer‐reviewed publications. Although this method is broadly comparable to the COSMIN approach, differences may have influenced the overall ratings.

Furthermore, although the review included individuals of any age, only one pediatric‐specific PROM (JAFI) was identified among the instruments used to evaluate foot orthoses. The limited number of child‐focused instruments and the relatively scarce psychometric evidence available for this population restrict the strength of conclusions that can be drawn regarding PROM use in pediatric patients.

Finally, although no restrictions on language were applied, most included studies were published in English, which may limit the generalizability of findings to PROMs validated in other linguistic and cultural contexts. In addition, this review was unable to assess cross‐cultural validity or measurement invariance of the included PROMs, which represents an important limitation for their use in multinational or cross‐cultural clinical trials.

## 5 Conclusions

Despite the limited evidence available for some psychometric properties, this review indicates that most validated foot‐ and ankle‐specific PROMs used to evaluate foot orthosis interventions demonstrate acceptable measurement properties. Among them, the FFI [17] and the FHSQ [18] were the most frequently applied, whereas the VISA‐A [21] showed the strongest overall measurement properties, followed by the FFI‐R [19] and the FAOS [22]. These instruments may therefore be useful in studies assessing foot orthoses.

When selecting a PROM, clinicians and researchers should carefully consider its measurement properties to minimize the risk of inaccurate assessment. Finally, there is a clear need to either develop and validate a PROM specifically designed for evaluating foot orthosis interventions or to further improve the measurement properties of the existing questionnaires identified in this review.

## Author Contributions

**Javier Jiménez‐Guillen:** conceptualization, data curation, investigation, methodology, resources, validation, writing – original draft, writing – review and Editing. **Natalia Tovaruela‐Carrión:** conceptualization, formal analysis, investigation, supervision, writing – original draft, writing – review and editing. **Marta María Moreno‐Fresco:** methodology, validation, writing – review and editing. **Alfonso Martínez‐Nova:** conceptualization, validation, writing – review and editing. **Pedro V. Munuera‐Martínez:** conceptualization, formal analysis, investigation, project administration, supervision, writing – review and editing.

## Funding

The authors have nothing to report.

## Ethics Statement

The authors have nothing to report.

## Consent

The authors have nothing to report.

## Conflicts of Interest

The authors declare no conflicts of interest.

## Supporting information


Supporting Information S1

## Data Availability

The data that support the findings of this study are available in the supplementary material of this article. The COSMIN user manual, the risk of bias scale, and the criteria for good psychometric properties are available on the official COSMIN website: https://www.cosmin.nl/.

## Appendix 1 Latest Searchs in the Databases

*First search*: PubMed (05/02/2025), Scopus (05/02/2025), Embase (06/02/2025), Cochrane Library (08/02/2025) y Web of Science (09/02/2025).

*Second search*: PubMed (29/04/2025), Scopus (29/04/2025), Embase (30/04/2025), Cochrane Library (30/04/2025) y Web of Science (01/05/2025). The last time the bibliography of the included articles was consulted was 12/05/2025.
