# Supplementary material for: Reporting and Utilization of Patient‐Reported Outcomes Measures in the Evaluation of Foot Orthoses Treatment: A Systematic Review
Source: J Foot Ankle Res. 2026 Mar 16;19(1):e70148. doi: 10.1002/jfa2.70148 (PMC13097690; doi:10.1002/jfa2.70148)
Supplement: Supplementary file 1 — Supporting Information S1 [file JFA2-19-e70148-s001.pdf]

SUPPLEMENTARY FILE 1

Online Resource 1 Search Strategies

First search: (The strategy followed is the same for each database, but it is adapted to the specific syntax and rules of each database)

PubMed

("patient reported outcome*" OR "quality of life" OR "surveys and questionnaires" OR "health status indicator*" OR "self report*" OR "diagnostic self evaluation" OR "outcome assess*" OR "outcome and process assessment, health care") AND ("foot orth*" OR splint* OR orthot* OR ((toe OR hallux*) AND (ortho* OR silicon*))) AND ("lower extremity" OR "lumbosacral region" OR pelvi* OR hip OR buttock* OR thigh* OR femur OR knee* OR patell* OR "genu valgum" OR "genu varum" OR leg OR tibi* OR "tarsal tunnel syndrome" OR fibula* OR "muscle, skeletal" OR ankle* OR foot* OR plantar* OR flatfoot OR "talipes cavus" OR gait* OR toe OR hallux* OR metatarsal* OR "forefoot, human" OR heel* OR metatarsus* OR "tarsal bones" OR "peroneal neuropathies" OR ligament* OR tendon*)

Filters: Type of article: Randomized controlled trial.

Embase

('patient-reported outcome*' OR 'quality of life'/exp OR 'quality of life' OR 'questionnaire*' OR 'health status indicator*' OR 'self report*' OR 'self evaluat*' OR 'outcome assess*' OR 'treatment outcome*') AND ('foot ortho*' OR 'splint*' OR 'orthosis*' OR (('toe'/exp OR 'toe' OR 'hallux*') AND ('orthosis*' OR 'silicone'/exp OR 'silicone'))) AND ('lower limb'/exp OR 'lower limb' OR 'lumbosacral region'/exp OR 'lumbosacral region' OR 'pelvi*' OR 'hip'/exp OR 'hip' OR 'buttock*' OR 'thigh*' OR 'femur'/exp OR 'femur' OR 'knee*' OR 'patell*' OR 'valgus knee'/exp OR 'valgus knee' OR 'varus knee'/exp OR 'varus knee' OR 'leg'/exp OR 'leg' OR 'tibi*' OR 'tibial nerve compression'/exp OR 'tibial nerve compression' OR 'fibula*' OR 'skeletal muscle'/exp OR 'skeletal muscle' OR 'ankle*' OR 'foot*' OR 'plantar*' OR 'flatfoot'/exp OR 'flatfoot' OR 'pes cavus'/exp OR 'pes cavus' OR 'gait*' OR 'toe'/exp OR 'toe' OR 'hallux*' OR 'metatarsal*' OR 'forefoot'/exp OR 'forefoot' OR 'heel*' OR 'metatarsal bone'/exp OR 'metatarsal bone' OR 'metatarsus*' OR 'tarsal bone'/exp OR 'tarsal bone' OR 'peroneal neuropathy'/exp OR 'peroneal neuropathy' OR 'ligament*' OR 'tendon*') AND [randomized controlled trial]/lim AND [article]/lim

Filters: Articles and randomized controlled trials. The “Search as broadly as posible” option was checked.

Scopus

TITLE-ABS-KEY-AUTH (("patient reported outcome*" OR "quality of life" OR "surveys and questionnaires" OR "health status indicator*" OR "self report*" OR "diagnostic self evaluation" OR "outcome assess*" OR "outcome and process assessment, health care") AND ("foot orth*" OR splint* OR orthot* OR ((toe OR hallux*) AND (ortho* OR silicon*))) AND ("lower extremity" OR "lumbosacral region" OR pelvi* OR hip OR buttock* OR thigh* OR femur OR knee* OR patell* OR "genu valgum" OR "genu varum" OR leg OR 2 tibi* OR "tarsal tunnel syndrome" OR fibula* OR "muscle, skeletal" OR ankle* OR foot* OR plantar* OR flatfoot OR "talipes cavus" OR gait* OR

toe OR hallux* OR metatarsal* OR "forefoot, human" OR heel* OR metatarsus* OR "tarsal bones" OR "peroneal neuropathies" OR ligament* OR tendon*) AND (random*)) DOCTYPE (ar)

Filters: DOCTYPE (ar) filter was added to the strategy to filter by articles. Also (random*) was added to be able to find randomized controlled trials.

Cochrane Library

((patient reported NEXT outcome*) OR "quality of life" OR "surveys and questionnaires" OR (health status NEXT indicator*) OR (self NEXT report*) OR "diagnostic self evaluation" OR (outcome NEXT assess*) OR "outcome and process assessment, health care") AND ((foot NEXT orth*) OR splint* OR orthot* OR ((toe OR hallux*) AND (ortho* OR silicon*))) AND ("lower extremity" OR "lumbosacral region" OR pelvi* OR hip OR buttock* OR thigh* OR femur OR knee* OR patell* OR "genu valgum" OR "genu varum" OR leg OR tibi* OR "tarsal tunnel syndrome" OR fibula* OR "muscle, skeletal" OR ankle* OR foot* OR plantar* OR flatfoot OR "talipes cavus" OR gait* OR toe OR hallux* OR metatarsal* OR "forefoot, human" OR heel* OR metatarsus* OR "tarsal bones" OR "peroneal neuropathies" OR ligament* OR tendon*)

Filters: Trials, All years, All dates and “search word variations” options were checked.

Web of Science

((ALL=(("patient reported outcome*" OR "quality of life" OR "surveys and questionnaires" OR "health status indicator*" OR "self report*" OR "diagnostic self evaluation" OR "outcome assess*" OR "outcome and process assessment, health care") AND ("foot orth*" OR splint* OR orthot* OR ((toe OR hallux*) AND (ortho* OR silicon*))) AND ("lower extremity" OR "lumbosacral region" OR pelvi* OR hip OR buttock* OR thigh* OR femur OR knee* OR patell* OR "genu valgum" OR "genu varum" OR leg OR tibi* OR "tarsal tunnel syndrome" OR fibula* OR "muscle, skeletal" OR ankle* OR foot* OR plantar* OR flatfoot OR "talipes cavus" OR gait* OR toe OR hallux* OR metatarsal* OR "forefoot, human" OR heel* OR metatarsus* OR "tarsal bones" OR "peroneal neuropathies" OR ligament* OR tendon*))) AND ALL=((random*))) AND DT=(Article)

Filters: DT=(Article) filter was added to the strategy to filter by articles. Also (random*) was added to be able to find randomized controlled trials.

Second search: (The strategy followed is the same for each database, but it is adapted to the specific syntax and rules of each database)

PubMed

("foot function index"[tiab] OR FFI[tiab] OR "revised foot function index"[tiab] OR "foot function index revised"[tiab] OR FFI-R[tiab] OR "FFI revised"[tiab] OR "revised 3 FFI"[tiab] OR "foot health status questionnaire"[tiab] OR FHSQ[tiab] OR "juvenile arthritis foot disability index"[tiab] OR JAFI[tiab] OR "manchester foot pain and disability index"[tiab] OR MFPDI[tiab] OR "victorian institute of sports assessment-achilles"[tiab] OR VISA-A[tiab] OR "VISA Achilles"[tiab] OR "foot and ankle outcome score"[tiab] OR FAOS[tiab] OR "visual analogue scale foot and ankle"[tiab] OR VAS-

FA[tiab] OR "VAS FA"[tiab] OR VASFA[tiab] OR "foot and ankle disability index"[tiab] OR FADI[tiab] OR "american orthopaedic foot and ankle society"[tiab] OR AOFAS[tiab]) AND (reproducibility[tiab] OR psychometrics[MeSH] OR psychometr*[tiab] OR reliability[tiab] OR validity[tiab] OR consistency[tiab] OR cronbach*[tiab] OR agreement[tiab] OR precision[tiab] OR test-retest[tiab] OR (test[tiab] AND retest[tiab]) OR stability[tiab] OR interrater[tiab] OR inter-rater[tiab] OR intrarater[tiab] OR intra-rater[tiab] OR intertester[tiab] OR inter-tester[tiab] OR intratester[tiab] OR intra-tester[tiab] OR interobserver[tiab] OR inter-observer[tiab] OR intraobserver[tiab] OR intra-observer[tiab] OR intertechnician[tiab] OR inter-technician[tiab] OR intratechnician[tiab] OR intra- technician[tiab] OR interexaminer[tiab] OR inter-examiner[tiab] OR intraexaminer[tiab] OR intra-examiner[tiab] OR interindividual[tiab] OR inter-individual[tiab] OR intraindividual[tiab] OR intra-individual[tiab] OR interparticipant[tiab] OR inter-participant[tiab] OR intraparticipant[tiab] OR intra-participant[tiab] OR kappa[tiab] OR kappa‘s[tiab] OR kappas[tiab] OR repeatability[tiab] OR replicability[tiab] OR concordance[tiab] OR intraclass[tiab] OR discriminative[tiab] OR discriminant[tiab] OR variability[tiab] OR sensitivity[tiab] OR responsiveness[tiab] OR interpretability[tiab] OR "ceiling effect"[tiab] OR "floor effect"[tiab] OR "Item response model"[tiab] OR IRT[tiab] OR Rasch[tiab] OR "Differential item functioning"[tiab] OR DIF[tiab] OR "computer adaptive testing"[tiab] OR "cross-cultural equivalence"[tiab])

Filters: No filter was checked.

<u>Embase</u>

('foot function index':ti,ab OR 'ffi':ti,ab OR 'revised foot function index':ti,ab OR 'foot function index revised':ti,ab OR 'ffi-r':ti,ab OR 'ffi revised':ti,ab OR 'revised ffi':ti,ab OR 'foot health status questionnaire':ti,ab OR 'fhsq':ti,ab OR 'juvenile arthritis foot disability index':ti,ab OR 'jafi':ti,ab OR 'manchester foot pain and disability index':ti,ab OR 'mfpdi':ti,ab OR 'victorian institute of sports assessment-achilles':ti,ab OR 'visa-a':ti,ab OR 'visa achilles':ti,ab OR 'foot and ankle outcome score':ti,ab OR 'faos':ti,ab OR 'visual analogue scale foot and ankle':ti,ab OR 'vas-fa':ti,ab OR 'vas fa':ti,ab OR 'vasfa':ti,ab OR 'foot and ankle disability index':ti,ab OR 'fadi':ti,ab OR 'american orthopaedic foot and ankle society':ti,ab OR 'aofas':ti,ab) AND ('reproducibility':ti,ab OR 'psychometry'/exp OR 'psychometry' OR 'psychometr*':ti,ab OR 'reliability':ti,ab OR 'validity':ti,ab OR 'consistency':ti,ab OR 'cronbach*':ti,ab OR 'agreement':ti,ab OR 'precision':ti,ab OR 'test-retest':ti,ab OR ('test':ti,ab AND 'retest':ti,ab) OR 'stability':ti,ab OR 'interrater':ti,ab OR 'inter- rater':ti,ab OR 'intrarater':ti,ab OR 'intra-rater':ti,ab OR 'intertester':ti,ab OR 'inter-tester':ti,ab OR 'intratester':ti,ab OR 'intra-tester':ti,ab OR 'interobserver':ti,ab OR 'inter-observer':ti,ab OR 'intraobserver':ti,ab OR 'intra-observer':ti,ab OR 'intertechnician':ti,ab OR 'inter- technician':ti,ab OR 'intratechnician':ti,ab OR 'intra-technician':ti,ab OR 'interexaminer':ti,ab OR 'inter-examiner':ti,ab OR 'intraexaminer':ti,ab OR 'intra-examiner':ti,ab OR 'interindividual':ti,ab OR 'inter-individual':ti,ab OR 'intraindividual':ti,ab OR 'intra- individual':ti,ab OR 'interparticipant':ti,ab OR 'inter-participant':ti,ab OR 'intraparticipant':ti,ab OR 'intra-participant':ti,ab OR 'kappa':ti,ab OR 'kappa`s':ti,ab OR 'kappas':ti,ab OR 'repeatability':ti,ab OR 'replicability':ti,ab OR 'concordance':ti,ab OR 'intraclass':ti,ab OR 'discriminative':ti,ab OR 'discriminant':ti,ab OR 'variability':ti,ab OR 'sensitivity':ti,ab OR 'responsiveness':ti,ab OR

'interpretability':ti,ab OR 'ceiling effect':ti,ab OR 'floor effect':ti,ab OR 'item response model':ti,ab OR 'irt':ti,ab OR 'rasch':ti,ab OR 'differential item functioning':ti,ab OR 'dif':ti,ab OR 'computer adaptive testing':ti,ab OR 'cross-cultural equivalence':ti,ab)

Filters: Articles. The “Search as broadly as possible” option was checked.

Scopus

TITLE-ABS(("foot function index" OR FFI OR "revised foot function index" OR "foot function index revised" OR FFI-R OR "FFI revised" OR "revised FFI" OR "foot health status questionnaire" OR FHSQ OR "juvenile arthritis foot disability index" OR JAFI OR "manchester foot pain and disability index" OR MFPDI OR "victorian institute of sports assessment-achilles" OR VISA-A OR "VISA Achilles" OR "foot and ankle outcome score" OR FAOS OR "visual analogue scale foot and ankle" OR VAS-FA OR "VAS FA" OR VASFA OR "foot and ankle disability index" OR FADI OR "american orthopaedic foot and ankle society" OR AOFAS) AND (reproducibility OR psychometr* OR reliability OR validity OR consistency OR cronbach* OR agreement OR precision OR test-retest OR (test AND retest) OR stability OR interrater OR inter-rater OR intrarater OR intra-rater OR intertester OR inter-tester OR intratester OR intra-tester OR interobserver OR inter-observer OR intraobserver OR intra-observer OR intertechnician OR inter-technician OR intratechnician OR intra-technician OR interexaminer OR inter-examiner OR intraexaminer OR intra-examiner OR interindividual OR inter-individual OR intraindividual OR intra- individual OR interparticipant OR inter-participant OR intraparticipant OR intra-participant OR kappa OR kappa's OR kappas OR repeatability OR replicability OR concordance OR intraclass OR discriminative OR discriminant OR variability OR sensitivity OR responsiveness OR interpretability OR "ceiling effect" OR "floor effect" OR "Item response model" OR IRT OR Rasch OR "Differential item functioning" OR DIF OR "computer adaptive testing" OR "cross-cultural equivalence"))

Filters: Articles.

Web of Science

TS=(("foot function index" OR FFI OR "revised foot function index" OR "foot function index revised" OR FFI-R OR "FFI revised" OR "revised FFI" OR "foot health status questionnaire" OR fhss OR "juvenile arthritis foot disability index" OR jami OR "manchester foot pain and disability index" OR mfedi OR "victorian institute of sports assessment- achilles" OR VISA-A OR "VISA Achilles" OR "foot and ankle outcome score" OR FAOS OR "visual analogue scale foot and ankle" OR VAS-FA OR "VAS FA" OR vaska OR "foot and ankle disability index" OR FADI OR "american orthopaedic foot and ankle society" OR AOFAS) AND (reproducibility OR psychometr* OR reliability OR validity OR consistency OR cronbach* OR agreement OR precision OR test-retest OR (test AND retest) OR stability OR interrater OR inter-rater OR intrarater OR intra-rater OR intertester OR inter-tester OR intratester OR intra-tester OR interobserver OR inter-observer OR intraobserver OR intra- observer OR intertechnicism OR inter-technician OR intertechnician OR intra-technician OR interexaminer OR inter-examiner OR intraexaminer OR intra-examiner OR interindividual OR inter-individual OR intraindividual OR intra-individual OR interparticipants OR inter- participant OR interparticipant OR intra-participant OR kappa OR kappa's OR kappas OR repeatability

OR replicability OR concordance OR intraclass OR discriminative OR discriminant OR variability OR sensitivity OR responsiveness OR interpretability OR "ceiling effect" OR "floor effect" OR "Item response model" OR IRT OR Rasch OR "Differential item functioning" OR DIF OR "computer adaptive testing" OR "cross-cultural equivalence"))

Filters: Articles.

<u>Cochrane Library</u>

("foot function index":ti,ab OR FFI:ti,ab OR "revised foot function index":ti,ab OR "foot function index revised":ti,ab OR FFI-R:ti,ab OR "FFI revised":ti,ab OR "revised FFI":ti,ab OR "foot health status questionnaire":ti,ab OR FHSQ:ti,ab OR "juvenile arthritis foot disability index":ti,ab OR JAFI:ti,ab OR "manchester foot pain and disability index":ti,ab OR MFPDI:ti,ab OR "victorian institute of sports assessment-achilles":ti,ab OR VISA-A:ti,ab OR "VISA Achilles":ti,ab OR "foot and ankle outcome score":ti,ab 5 OR FAOS:ti,ab OR "visual analogue scale foot and ankle":ti,ab OR VAS-FA:ti,ab OR "VAS FA":ti,ab OR VASFA:ti,ab OR "foot and ankle disability index":ti,ab OR FADI:ti,ab OR "american orthopaedic foot and ankle society":ti,ab OR AOFAS:ti,ab) AND (reproducibility:ti,ab OR psychometrics:ti,ab OR psychometr*:ti,ab OR reliability:ti,ab OR validity:ti,ab OR consistency:ti,ab OR cronbach*:ti,ab OR agreement:ti,ab OR precision:ti,ab OR test-retest:ti,ab OR (test:ti,ab AND retest:ti,ab) OR stability:ti,ab OR interrater:ti,ab OR inter-rater:ti,ab OR intrarater:ti,ab OR intra-rater:ti,ab OR intertester:ti,ab OR inter-tester:ti,ab OR intratester:ti,ab OR intra-tester:ti,ab OR interobserver:ti,ab OR inter-observer:ti,ab OR intraobserver:ti,ab OR intra- observer:ti,ab OR intertechnician:ti,ab OR inter-technician:ti,ab OR intratechnician:ti,ab OR intra-technician:ti,ab OR interexaminer:ti,ab OR inter-examiner:ti,ab OR intraexaminer:ti,ab OR intra-examiner:ti,ab OR interindividual:ti,ab OR inter-individual:ti,ab OR intraindividual:ti,ab OR intra-individual:ti,ab OR interparticipant:ti,ab OR inter- participant:ti,ab OR intraparticipant:ti,ab OR intra-participant:ti,ab OR kappa:ti,ab OR "kappa‘s":ti,ab OR kappas:ti,ab OR repeatability:ti,ab OR replicability:ti,ab OR concordance:ti,ab OR intraclass:ti,ab OR discriminative:ti,ab OR discriminant:ti,ab OR variability:ti,ab OR sensitivity:ti,ab OR responsiveness:ti,ab OR interpretability:ti,ab OR "ceiling effect":ti,ab OR "floor effect":ti,ab OR "item response model":ti,ab OR IRT:ti,ab OR Rasch:ti,ab OR "differential item functioning":ti,ab OR DIF:ti,ab OR "computer adaptive testing":ti,ab OR "cross-cultural equivalence":ti,ab)

Filters: All dates and “search word variations” options were checked.

SUPPLEMENTARY FILE 2

Online Resource 2 Total PROMs identified

Table 5. Total PROMs identified in the articles included of the first search

PROM	References
**1. Visual Analogue Scale (VAS)**	Alfaro Santafé et al. (2021) [42]; Andreasen et al. (2013) [43]; Asgaonkar y Kadam, (2013) [44]; Bari et al. (2024) [45]; Bishop et al. (2018) [46]; Burns et al. (2009) [47]; Çaglar Okur & Aydin, (2019) [48]; Campos et al. (2015) [49]; Casado Hernández et al. (2018) [50]; Castro Méndez et al. (2012) [51]; Castro Méndez et al. (2021) [52]; Cho et al. (2009) [53]; Coda et al. (2014) [54]; Collins et al. (2008) [55]; De Oliveira et al. (2019) [56]; Fellas et al. (2022) [57]; Ferreira et al. (2021) [58]; Greitemann et al. (2012) [59]; Hellstrand Tang et al. (2014) [60]; Jones et al. (2013) [61]; Kelly & Winson, (1998) [62]; Lewinson et al. (2015) [63]; McRitchie & Curran, (2007) [64]; Menz et al. (2017) [65]; Mills et al. (2012) [66]; Moreira et al. (2016) [67]; O´Sullivan et al. (2021) [68]; Oliveira et al. (2015) [69]; Parashar & Kuman, (2025) [70]; Rannisto et al. (2019) [71]; Reina-Bueno et al. (2019) [2]; Robert-Lachaine et al. (2024) [72]; Segal et al. (2009) [73]; Shim et al. (2021) [74]; Simon et al. (2025) [75]; Tan et al. (2019) [76]; Taseh et al. (2024) [77]; Toda & Tsukimura, (2024) [78]; Torkki et al. (2001) [79]; Van Raaij et al. (2010) [80]; Vicenzino et al. (2008) [81]; Walther et al. (2013) [82]; Wegener et al. (2016) [83]; Whittaker et al. (2019) [84]; Wrobel et al. (2015) [85]; Wyndowet al. (2021) [86]; Yildiz et al. (2022) [87]; Yucel et al. (2013) [88]; Yurt et al. (2019) [89]
**2. Western Ontario and McMaster Universities Osteoarthritis Index**	Baker et al. (2007) [90]; Barati et al. (2022) [91]; Barrios et al. (2009) [92]; Bennell et al. (2011) [93]; Campos et al. (2015) [49]; Jones et al. (2013) [61]; Maillefert et al. (2001) [94]; Rafiaee & Karimi, (2012) [95]; Robert-Lachaine et al. (2024) [72]; VanRaaij et al. (2010) [80]
**3. Foot Function Index (FFI) ***	Baldassin et al. (2009) [96]; Çaglar Okur & Aydin, (2019) [48]; Cho et al. (2009) [53]; De Oliveira et al. (2019) [56]; Gatt et al. (2016) [97]; Maddali Bongi et al. (2014) [98]; Moreira et al. (2016) [67]; Oliveira et al. (2015) [69]; Palomo-Toucedo et al. (2019) [99]; Powell et al. (2005) [100]; Rasenberg et al. (2021) [101]; Reina-Bueno et al. (2019) [2]; Rome et al. (2017) [102]; Shim et al. (2021) [74]; Yurt et al. (2019) [89]
**4. Modified Quebec Questionnaire for Evaluating User Satisfaction with Assistive Technology (QUEST)**	Bari et al. (2024) [45]

Table 5 (continuation)

PROM	References
**5. Numeric Rating Scale (NRS)**	Bennell et al. (2011) [93]; Cambron et al. (2017) [103]; Felson et al. (2019) [104]; Halstead et al. (2016) [105]; Heide et al. (2024) [106]; Hunter et al. (2023) [107]; Palomo-Toucedo et al. (2019) [99]; Paterson et al. (2022) [108]; Rasenberg et al. (2021) [101]; Sadler et al. (2023) [109]; Schmitt et al. (2025) [110]; Schwarze et al. (2021) [111]; Trotter & Pierrynowski, (2008) [1]
**6. Quality of Life Evaluation Instrument (AQoL)**	Bennell et al. (2011) [93]; Paterson et al. (2022) [108]
**7. Foot Health Status Questionnaire (FHSQ)***	Burns et al. (2006) [112]; Burns et al. (2009) [47]; Çaglar Okur & Aydin, (2019) [48]; Crosbie & Burns, (2007) [113]; De Oliveira et al. (2019) [56]; Landorf et al. (2006) [114]; McRitchie & Curran, (2007) [64]; Menz et al. (2017) [65]; Moreira et al. (2016) [67]; Munteanu et al. (2021) [115]; Oliveira et al. (2015) [69]; Paterson et al. (2022) [108]; Rome et al. (2017) [102]; Whittaker et al. (2019) [84]
**8. Physical Activity Scale for Older Adults (PASE)**	Bennell et al. (2011) [93]; Lewinson et al. (2016) [116]; Paterson et al. (2022) [108]
**9. Functional Index Questionnaire (FIQ)**	Collins et al. (2008) [55]; Vicenzino et al. (2008) [81]
**10. Lower Extremity Functional Scale (LEFS)**	Botelho et al. (2022) [117]; Vicenzino et al. (2008) [81]
**11. Medical Outcomes Study. 36-Items short form (SF-36)**	Botelho et al. (2022) [117]; Burns et al. (2006) [112]; Burns et al. (2009) [47]; Crosbie & Burns, (2007) [113]; De Oliveira et al. (2019) [56]; Greitemann et al. (2012) [59]; Moreira et al. (2016) [67]; Munteanu et al. (2015) [23]; Oliveira et al. (2015) [69]; Segal et al. (2009) [73]; Sharifian et al. (2018) [118]; Shim et al. (2021)[74]; Vicenzino et al. (2008) [81]; Whittaker et al. (2019) [84]; Wrobel et al. (2015) [85]; Wyndow et al. (2021) [86]; Yurt et al. (2019) [89]
**12. Patient Specific Functional Scale (PSFS)**	Burns et al. (2009) [47]; Mills et al. (2012) [66]; Vicenzino et al. (2008) [81]

Table 5 (continuation)

PROM	References
**13. Foot Function Index Revised (FFI-R) ***	Çaglar Okur & Aydin, (2019) [48]; Heide et al. (2024) [106]; Menz et al. (2017) [65]; Wrobel et al. (2015) [85]
**14. Lequesne Index**	Campos et al. (2015) [49]; Toda & Tsukimura, (2024) [78]
**15. Pediatric Quality of Life Inventory (PedsQL 4.0)**	Coda et al. (2014) [54]; Hsieh et al. (2018) [119]; Powell et al. (2005) [100]
**16. Pediatric Quality of Life Inventory. Rheumatology Module (PedsQL 3.0)**	Coda et al. (2014) [54]; Fellas et al. (2022) [57]
**17. Owestry Disability Index (ODI)**	Cambron et al. (2017) [103]; Castro Méndez et al. (2012) [51]; Castro Méndez et al. (2021) [52]; Rannisto et al. (2019) [71]; Sadler et al. (2023) [109]
**18. Kujala Anterior Knee Pain Scale**	Collins et al. (2008) [55]; Mills et al. (2012) [66]; O´Sullivan et al. (2021) [68]; Simon et al. (2025) [75]; Tan et al. (2019) [76]; Vicenzino et al. (2008) [81]; Wyndow et al. (2021) [86]
**19. International Knee Documentation Committee Subjective Knee Assessment Form (IKDC score)**	Dammerer et al. (2019) [120]
**20. Knee and osteoarthritis outcomes score (KOOS)**	Dammerer et al. (2019) [120]; Dwakaranathan et al. (2022) [121]; Felson et al. (2019) [104]; Ferreira et al. (2021) [58]; Lewinson et al. (2016) [116]; Mahmoodi et al. (2024) [122]; Robert-Lachaine et al. (2024) [72]; Salam et al. (2019) [123]; Segal et al. (2009) [73]; Tan et al. (2019) [76]
**21. MARX score**	Dammerer et al. (2019) [120]

Table 5 (continuation)

PROM	References
**22. Medical Outcomes Study. 12-ítems short form (SF-12)**	Dammerer et al. (2019); Menz et al. (2017) [65]; Munteanu et al. (2021) [115]; Rasenberg et al. (2021) [101]; Reina-Bueno et al. (2019) [2]
**23. Nordic Musculoskeletal Questionnaire (NMQ)**	De Almeida et al. (2016) [124]
**24. Juvenil Arthritis Foot Disability Index (JAFI) ***	Fellas et al. (2022) [57]
**25. American Orthopaedic Foot and Ankle Society. Clinical Rating Scale for Foot and Ankle (AOFAS-AH) ***	Grim et al. (2019) [125]; Yildiz et al. (2022) [87]
**26. Foot Pain and Function Scale (FPFS)**	Grim et al. (2019) [125]
**27. Visual Analogue Scale-Foot and Ankle (VAS-FA) ***	Grim et al. (2019) [125]
**28. Patient Global Impression Change (PGIC)**	Halstead et al. (2016) [105]; Heide et al. (2024) [106]; Mills et al. (2012) [66]
**29. Manchester Foot Pain and Disability Index (MFPDI) ***	Halstead et al. (2016) [105]; Palomo-Toucedo et al. (2023) [99]; Reina-Bueno et al. (2019) [2]
**30. Multiple Sclerosis Impact Scale**	Hatton et al. (2023) [127]
**31. Walking Scale for Multiple Sclerosis**	Hatton et al. (2023) [127]
**32. Quality of Life Instrument for Multiple Sclerosis**	Hatton et al. (2023) [127]

Table 5 (continuation)

PROM	References
**33. Modified Fatigue Impact Scale**	Hatton et al. (2023) [127]
**34. Medical Outcomes Study Pain Effects Scale**	Hatton et al. (2023) [127]
**35. Perceived Deficits Questionnaire**	Hatton et al. (2023) [127]
**36. Falls Efficacy Scale-International**	Hatton et al. (2023) [127]
**37. Health Status Inventory (RAND-12)**	Heide et al. (2024) [106]
**38. Pediatric Outcomes Data Collection Instrument (PODCI)**	Hsieh et al. (2024) [119]
**39. Hip Ostheoarthritis Outcome Score-12 items (HOOS-12)**	King et al. (2024) [127]
**40. Brief Fear of Movement Scale for Osteo-arthritis**	King et al. (2024) [127]
**41. Patient Health Questionnaire (PHQ-9)**	King et al. (2024) [127]
**42. Global Rating of Change (GROC)**	King et al. (2024) [127]; O´Sullivan et al. (2021) [68]; Tan et al. (2019) [76]; Wyndow et al. (2021) [86]
**43. Credibility and Expectancy Questionnaire (CEQ)**	King et al. (2024) [127]; Menz et al. (2017) [65]; O´Sullivan et al. (2021) [68]; Paterson et al. (2022) [108]; Tan et al. (2019) [76]
**44. UCLA Activity Scale**	Lewinson et al. (2016) [116]
**45. Incidental and Planned Activities Questionnaire**	Menz et al. (2017) [65]

Table 5 (continuation)

PROM	References
**46. International Physical Activity Questionnaire (IPAQ)**	Munteanu et al. (2021) [115]
**47. Health Assessment Questionnaire (HAQ)**	Moreira et al. (2016) [67]
**48. Victorian Institute of Sport Assessment-Achilles tendon questionnaire (VISA-A) ***	Munteanu et al. (2015) [23
**49. Patient Perception of Treatment Effectiveness**	Munteanu et al. (2015) [23]
**50. Physical Activity Level of the Previous Week (7 day Physical Activity Recall Questionnaire)**	Munteanu et al. (2015) [23]; Vicenzino et al. (2008) [81]; Whittaker et al. (2019) [84]
**51. Foot and Ankle Outcomes Score (FAOS) ***	Nakhaee et al. (2023) [3]; Norouzi et al. (2023) [128]; Roos et al. (2006) [129]; Sharifian et al. (2018) [118]; Shim et al. (2021) [74]; Schwarze et al. (2021) [111]; Taseh et al. (2024) [77]
**52. European Quality of Life Questionnaire (EQ-5D-5L)**	Munteanu et al. (2021) [115]; O´Sullivan et al. (2021) [68]; Parker et al. (2019) [130]; Rome et al. (2004) [131]; Rome et al. (2017) [102]; Schmitt et al. (2025) [110]
**53. Global Effect Perceived by the Patient**	Moreira et al. (2016) [67]; Vicenzino et al. (2008) [81]
**54. Global Improvement Scale**	Mills et al. (2012) [66]
**55. Knee Injuries and Osteoarthritis Outcome Score (KOOS-child)**	O´Sullivan et al. (2021) [68]
**56. Knee Injuries and Osteoarthritis Outcome Score-Patellofemoral Subscale (KOOS-PF)**	O´Sullivan et al. (2021) [68]; Wyndow et al. (2021) [86]

Table 5 (continuation)

PROM	References
**57. Acceptable Sympton Status of the Patient**	O´Sullivan et al. (2021) [68]
**58. Capacity Measuring Instrument for Adults (ICECAP-A)**	Parker et al. (2019) [130]
**59. Patient Perceived Satisfactory Improvement (PPSI)**	Paterson et al. (2022) [108]
**60. Pediatric Pain Questionnaire-Visual Analogue Scale**	Powell et al. (2005) [100]
**61. Health-related Quality of Life (RAND-36)**	Rannisto et al. (2019) [71]
**62. Roland Morris Disability Questionnaire**	Rannisto et al. (2019) [71], Rosner et al. (2014) [132]
**63. Short Questionnaire to Assess Health-Beneficial Physical Activity (SQUASH Questionnaire)**	Rasenberg et al. (2021) [101]
**64. Quadruple Numeric Pain Rating Scale**	Rosner et al. (2014) [132]
**65. International Physical Activity Questionnaire (IPAQ-short form 7)**	Sadler et al. (2023) [109]; Wyndow et al. (2021) [86]; Yurt et al. (2019) [89]
**66. Foot and Ankle Disability Index (FADI) ***	Schmitt et al. (2025) [110]
**67. Oxford Knee Score (OKS)**	Schwarze et al. (2021) [111]

Table 5 (continuation)

PROM	References
**68. American Knee Society Clinical Rating System (AKSS)**	Schwarze et al. (2021) [111]
**69. Hannover Functional Capacity Questionnaire-Osteoarthritis and Pain of Knee**	Schwarze et al. (2021) [111]
**70. American Orthopaedic Foot and Ankle Society. Clinical Rating System for the Metatarsophalan-geal and Interphalangeal Joints of the Hallux (AOFAS-HJ) ***	Torkki et al. (2001) [79]
**71. Karlsson-Peterson Score (KP score)**	Shim et al. (2021) [74]
**72. Patient Reported Outcome Measurement Information System (PROMIS v1.0 – Pain Intensity 3a)**	Taseh et al. (2024) [77]
**73. Health-related Quality of Life Index (15-D)**	Torkki et al. (2001) [79]
**74. McGill Pain Questionnaire**	Vicenzino et al. (2008) [81]
**75. Hospital Anxiety and Depression Scale (HADS)**	Vicenzino et al. (2008) [81]; Wyndow et al. (2021) [86]
**76. Children´s version of the Varni Thompson Pediatric Pain Questionnaire**	Whitford & Esterman, (2007) [133]
**77. Children´s self-percepcion profile (SPPC)**	Whitford & Esterman, (2007) [133]

Table 5 (continuation)

PROM	References
**78. Fear Avoidance Component Scale (FACS)**	Whittaker et al. (2019) [84]
**79. PainDETECT Questionnaire**	Wyndow et al. (2021) [86]
**80. Tampa Scale for the Kinesiophobia (TSK)**	Wyndow et al. (2021) [86]
**81. Arthritis Self-Efficacy Scale (ASES)**	Wyndow et al. (2021) [86]
**82. World Health Organization Quality of Life Questionnaire (WHOQOL - BREF)**	Yildiz et al. (2022) [87]

* Validated and specific PROMS for foot and ankle

SUPPLEMENTARY FILE 3

Online Resource 3 Methodological quality and psychometric evidence scores for included articles of the second search

Table 6. Detailed scores of the COSMIN risk of bias scale and criteria for good psychometric properties

	Content validity		Structural validity		Internal consistency		Reliability		Measurement error		Criterion validity		Construct validity				Responsiveness	
	MQ	E	MQ	E	MQ	E	MQ	E	MQ	E	MQ	E	C		D		MQ	E
													MQ	E	MQ	E		
**<u>Nº 1: FFI</u>**																		
Amri et al. (2022)	I	+	D	+	VG	+	A	+					A	+				
Anjum et al. (2024)	I	+			I	?	A	+	I	?			VG	+				
Bovonsun-thonchai et al. (2020)	D	?	I	-	VG	+	A	+			I	?	A	+				
Budiman-Mak et al. (1991)	I	-	D	?	VG	+	A	+					D	+			I	?
González-Sán-chez et al. (2018)	D	?	I	-	I	?	A	+	I	?	I	?	I	?				
Huh et al. (2016)	D	?			I	?	A	+					VG	+				
Jorgensen et al. (2015)	D	+			I	?	A	+										
Khan et al. (2022)	D	?			I	?	VG	+					VG	+				

Table 6 (continuation)

	Content validity		Structural validity		Internal consistency		Reliability		Measurement error		Criterion validity		Construct validity				Responsiveness	
													C		D			
	MQ	E	MQ	E	MQ	E	MQ	E	MQ	E	MQ	E	MQ	E	MQ	E	MQ	E
Kuyvenhoven et al. (2002)	D	-	D	+	VG	+	D	+									I	?
Martinelli et al. (2014)	I	?			I	?	I	+	I	?			D	+			I	?
Martinez et al. (2016)	I	?			I	?	D	+	I	?			I	?				
Mousavian et al. (2019)	I	?			I	?	D	+					I	?				
Naal et al. (2008)	D	+			I	?	I	+	I	?			I	?				
Paez-Moguer et al. (2014)	I	?	I	-	I	?							I	?				
Pourtier-Piotte et al. (2015)	D	?	I	-	I	?	D	+	I	?			I	?			I	?
Sidiq et al. (2024)	D	?	D	-	VG	+	A	+	I	?			VG	+				
Srimakarat et al. (2018)	D	?			I	?	D	+					I	?				

Table 6 (continuation)

	Content validity		Structural validity		Internal consistency		Reliability		Measurement error		Criterion validity		Construct validity				Responsiveness	
	MQ	E	MQ	E	MQ	E	MQ	E	MQ	E	MQ	E	C		D		MQ	E
													MQ	E	MQ	E		
Vetrano et al. (2014)	I	-			I	?	A	+										
Wu et al. (2008)	D	+			I	?	I	+					I	?				
Yaliman et al. (2014)	D	?																
Yi et al. (2015)	D	+																
**<u>Nº 2: FHSQ</u>**																		
Alshammari et al. (2023)	I	+	A	+	VG	+	A	+	I	?			VG	+				
Bidari et al. (2021)	I	?			I	?	A	+			I	?						
Bennett et al. (1998)	I	?	D	+	VG	+	A	+			I	?	I	?	I	?		
Cuesta-Vargas et al. (2013)	D	?	A	+	VG	+	A	+			I	?	I	?				
Ferreira et al. (2008)	D	?			I	?	I	?					I	?				

Table 6 (continuation)

	Content validity		Structural validity		Internal consistency		Reliability		Measurement error		Criterion validity		Construct validity				Responsiveness	
	MQ	E	MQ	E	MQ	E	MQ	E	MQ	E	MQ	E	C		D		MQ	E
													MQ	E	MQ	E		
Martijn et al. (2023)	D	?			I	?	I	-	I	?			VG	+				
Riel et al. (2019)	D	+											I	?				
**<u>Nº 3: FFI-R</u>**																		
Budiman-Mak et al. (2006)	I	-	D	-	VG	+							I	?				
Mork et al. (2022)	D	+	I	-	I	+	A	+	A	+			A	+			VG	+
Rutkowski et al. (2017)	D	?			I	?	I	+					I	?	I	?		
Stéfani et al. (2017)	D	?			I	?	D	-										
Yagci et al. (2020)	I	?			I	?	A	+					I	?				
Yi et al. (2017)	I	+																
**<u>Nº 4: JAFI</u>**																		
André et al. (2004)	I	+			I	?	A	+					VG	+	I	?		

Table 6 (continuation)

	Content validity		Structural validity		Internal consistency		Reliability		Measurement error		Criterion validity		Construct validity				Responsiveness	
	MQ	E	MQ	E	MQ	E	MQ	E	MQ	E	MQ	E	C		D		MQ	E
													MQ	E	MQ	E		
**N° 5: MFPDI**																		
Azadinia et al. (2022)	I	+			I	?	A	+	I	?			VG	+				
Erh et al. (2019)	I	+	A	?	VG	+	D	+					VG	+				
Ferrari et al. (2008)	I	?	I	-	I	?	A	+					I	?				
Garrow et al. (2000)	D	+	D	+	I	?					I	?	I	?	I	?		
Gijón-Nogue-rón et al. (2014)	I	?	VG	+	VG	+												
Kaoulla et al. (2008)-	I	?	D	+	D	-							I	?				
Pedersen et al. (2013)	I	?					I	+					VG	+				
Van der Zwaard et al. (2014)	D	?	D	+	D	-	I	-	I	?			VG	+			VG	-

Table 6 (continuation)

	Content validity		Structural validity		Internal consistency		Reliability		Measurement error		Criterion validity		Construct validity				Responsiveness	
													C		D			
	MQ	E	MQ	E	MQ	E	MQ	E	MQ	E	MQ	E	MQ	E	MQ	E	MQ	E
**<u>Nº 6: VISA-A</u>**																		
Alshewaier et al. (2024)	I	+			I	?	A	+					D	+	A	+		
Bahari et al. (2022)	I	+	A	+	VG	+	A	+	I	?	I	?	I	?	I	?		
Chang et al. (2021)	I	?	A	+	VG	+	A	+	I	?			VG	+	VG	+		
De Mesquita et al. (2018)	I	?			I	?	A	+	I	?			VG	+	I	?		
Dogramaci et al. (2011)	D	+			I	-	I	+					I	?	I	?		
Hernández-Sánchez et al. (2018)	D	?	VG	?	VG	+	A	+	I	?			VG	+	I	?	I	?
Robinson et al. (2001)	D	?					A	+					I	?	I	?		
Iversen et al. (2016)	D	+			I	?	D	+			I	?			I	?	I	?
Jabbar et al. (2024)	D	+	D	+	VG	+	A	+	I	?			I	?	I	?		

Table 6 (continuation)

	Content validity		Structural validity		Internal consistency		Reliability		Measurement error		Criterion validity		Construct validity				Responsiveness	
	MQ	E	MQ	E	MQ	E	MQ	E	MQ	E	MQ	E	C		D		MQ	E
													MQ	E	MQ	E		
Kaux, Delvaux, Oppong-Kyei, Beaudart et al. (2016)	D	?			I	?	D	+					I	?	I	?		
Kaux, Delvaux, Oppong-Kyei, Dardenne et al. (2016)	D	?			I	?	D	+					D	+	D	+		
Keller et al. (2018)	D	?					A	+					I	?	I	?		
Ko et al. (2022)	I	?					A	+					I	?	I	?		
Lohrer y Nauk, (2009)	D	+			I	?	D	+					I	?	I	?		
Maffulli et al. (2008)	I	?			I	?	I	+					I	?				
Sierevelt et al. (2018)	D	?			I	?	A	+	I	?			D	+				
Silbernagel et al. (2005)	D	+	D	?	VG	+	D	+			I	?	I	?	I	?		

Table 6 (continuation)

	Content validity		Structural validity		Internal consistency		Reliability		Measurement error		Criterion validity		Construct validity				Responsiveness	
													C		D			
	MQ	E	MQ	E	MQ	E	MQ	E	MQ	E	MQ	E	MQ	E	MQ	E	MQ	E
Tu et al. (2022)	D	?	I	-	I	?	D	+			I	?	I	?	I	?	D	+
**<u>Nº 7: FAOS</u>**																		
Aditya et al. (2021)	I	?	I	-	I	?	D	+					VG	+			D	+
Angthong, (2016)	D	+			I	?	D	-					I	?				
Göksel Karatepe et al. (2009)	I	?			I	?	A	+					I	?				
Imoto et al. (2009)	D	?			I	?	D	+					I	?				
Larsen et al. (2017)	I	?					D	+	I	?								
Lee et al. (2013)	D	+	D	+	VG	+	D	+					I	?				
Ling et al. (2018)	D	+			I	?	A	+					I	?				
Negahban et al. (2010)	I	?			I	?	I	+	I	?			D	-				

Table 6 (continuation)

	Content validity		Structural validity		Internal consistency		Reliability		Measurement error		Criterion validity		Construct validity				Responsiveness	
	MQ	E	MQ	E	MQ	E	MQ	E	MQ	E	MQ	E	C		D		MQ	E
													MQ	E	MQ	E		
Navarro Flores et al. (2020)	I	?			I	?	I	+	I	?								
Pellegrini et al. (2020)	D	?	D	?	VG	+							VG	+	I	?		
Roos et al. (2001)	D	?	A	+	VG	+	I	+					D	+				
Sierevelt et al. (2015)	D	?	I	?	D	+	I	+	I	?			VG	+				
Tapaninaho et al. (2022)	I	-	D	-														
Van Bergen et al. (2014)	D	?			I	?	A	+	I	?			VG	+				
Van Den Akker-Scheekde et al. (2013)	D	?			I	?	A	+	I	?			I	?			I	?
<u>**Nº 8: VAS-FA**</u>																		
Angthong et al. (2011)	D	+			I	?	D	-					I	?				

Table 6 (continuation)

	Content validity		Structural validity		Internal consistency		Reliability		Measurement error		Criterion validity		Construct validity				Responsiveness	
													C		D			
	MQ	E	MQ	E	MQ	E	MQ	E	MQ	E	MQ	E	MQ	E	MQ	E	MQ	E
Cervera-Garvi et al. (2024)	D	-	D	-	VG	+	A	+	A	+			I	?				
Gur et al. (2017)	D	?			I	?	D	+					I	?	I	?		
Kovaleva et al. (2023)	I	?			I	?	D	+	D	+	I	?					I	?
Nair et al. (2015)													I	?			I	?
Richter et al. (2006)	D	?											I	?				
Repo et al. (2018)	D	?	D	-	VG	+	D	+			I	?	A	+				
Zarei et al. (2025)	I	+			I	?	A	+					I	?	I	?		
<u>**Nº 9: FADI**</u>																		
Akulaev et al. (2023)	I	?	I	-	I	?	A	+			I	?	D	+	I	?	I	?
Eun et al. (2024)	I	?			I	?	A	+					I	?				

Table 6 (continuation)

	Content validity		Structural validity		Internal consistency		Reliability		Measurement error		Criterion validity		Construct validity				Responsiveness	
	MQ	E	MQ	E	MQ	E	MQ	E	MQ	E	MQ	E	C		D		MQ	E
													MQ	E	MQ	E		
Hale y Hertel, (2005)							I	+									I	?
Leigheb et al. (2020)	D	+					A	+					I	?				
Martin et al. (1999)																		
Popli et al. (2024)	I	?			I	?	D	+	I	?								
**<u>Nº 10: AOFAS-AH</u>**																		
Kitaoka y Sanders, (1994)																		
Alhadhoud et al. (2020)	I	+			I	?	A	+					I	?			I	?
Analay Akba-ba et al. (2016)	I	+			I	?	VG	+	I	?			I	?				
De Boer et al. (2017)	I	?	A	-	I	?	A	+	I	?			VG	+			VG	+
Ercihsen et al. (2020)	I	+			I	?	D	+	D	+			VG	+			VG	-

Table 6 (continuation)

	Content validity		Structural validity		Internal consistency		Reliability		Measurement error		Criterion validity		Construct validity				Responsiveness	
													C		D			
	MQ	E	MQ	E	MQ	E	MQ	E	MQ	E	MQ	E	MQ	E	MQ	E	MQ	E
Formichev et al. (2023)	D	?			I	?	I	+	I	?			VG	+			I	?
Kostuj et al. (2014)	D	?					D	+					I	?	I	?		
Leigheb et al. (2016)	D	+					A	+					I	?				
Makmetova et al. (2024)	D	?			I	?	A	+	I	?			VG	+				
Rodrigues et al. (2008)	D	?					VG	+					I	?				
Seyed Hoseinian et al. (2018)	I	?			I	?	D	-					I	?				
Vosoughi et al. (2018)	I	?			I	?	A	+					I	?				
<u>**Nº 11: AOFAS-HJ**</u>																		
Kitaoka y Sanders, (1994)																		
Alhadhoud et al. (2020)	I	+			I	?	A	+					I	?			I	?

Table 6 (continuation)

	Content validity		Structural validity		Internal consistency		Reliability		Measurement error		Criterion validity		Construct validity				Responsiveness	
	MQ	E	MQ	E	MQ	E	MQ	E	MQ	E	MQ	E	C		D		MQ	E
													MQ	E	MQ	E		
Kalaycioglu et al. (2023)	D	+			I	?	D	+	I	?			VG	+				
Leigheb et al. (2019)	D	+					A	+					I	?				
Mahdaviazad et al. (2020)	I	?			I	?	A	+					I	?				
Molano Castro et al. (2023)	I	?			I	?	A	+					I	?			I	?
Ziroglu et al. (2023)	I	+			I	?	A	+	I	?			I	?				

MQ=Methodological Quality E=Pyschometric evidence C=Convergent D=Discriminative I=Inadequate D=Doubtful A=Adequate VG=Very Good

LIST OF ALL REFERENCES FOUND IN THE SEARCHES

1. Trotter, L. C., & Pierrynowski, M. R. (2008). The short-term effectiveness of full-contact custom-made foot orthoses and prefabricated shoe inserts on lower-extremity musculoskeletal pain. *Journal of the American Podiatric Medical Association*, *98*(5), 357-363. https://doi.org/10.7547/0980357

2. Reina-Bueno, M., Vázquez-Bautista, M. D. C., Pérez-García, S., Rosende-Bautista, C., Sáez-Díaz, A., & Munuera-Martínez, P. V. (2019). Effectiveness of custom-made foot orthoses in patients with rheumatoid arthritis: A randomized controlled trial. *Clinical Rehabilitation*, *33*(4), 661-669. https://doi.org/10.1177/0269215518819118

3. Nakhaee, M., Mohseni-Bandpei, M., Mousavi, M. E., Shakourirad, A., Safari, R., Kashani, R. V., Mimar, R., Amiri, H., & Nakhaei, M. (2023). The effects of a custom foot orthosis on dynamic plantar pressure in patients with chronic plantar fasciitis: A randomized controlled trial. *Prosthetics and Orthotics International*, *47*(3), 241-252. https://doi.org/10.1097/PXR.0000000000000179

4. Jia, Y., Huang, H., & Gagnier, J. J. (2017). A systematic review of measurement properties of patient-reported outcome measures for use in patients with foot or ankle diseases. *Quality of Life Research*, *26*(8), 1969-2010. https://doi.org/10.1007/s11136-017-1542-4

5. Mokkink, L. B., Elsman, E. B., & Terwee, C. B. (2024). COSMIN guideline for systematic reviews of patient-reported outcome measures version 2.0. *Quality of Life Research*, *33*, 2929–2939. https://doi.org/10.1007/s11136-024-03761-6

6. Anjum, A., Tauqeer, S., Arooj, A., Javed, H., Shakeel, H., & Ikram, A. (2024). Translation, cross-cultural adaptation and validation of psychometric properties of Foot Function Index in Urdu-speaking population with ankle and foot disorders. *BMC Musculoskeletal Disorders*, *25*, 751. https://doi.org/10.1186/s12891-024-07857-5

7. Elsman, E. B. M., Mokkink, L. B., Terwee, C. B., Beaton, D., Gagnier, J. J., Tricco, A. C., Baba, A., Butcher, N. J., Smith, M., Hofstetter, C., Aiyegbusi, O. L., Berardi, A., Farmer, J., Haywood, K. L., Krause, K. R., Markham, S., Mayo-Wilson, E., Mehdipour, A., Ricketts, J., . . . Offringa, M. (2024). Guideline for reporting systematic reviews of outcome measurement instruments (OMIs): PRISMA-COSMIN for OMIs 2024. *Quality of Life Research*, *33*, 2029-2046. https://doi.org/10.1007/s11136-024-03634-y

8. Terwee, C. B., Jansma, E. P., Riphagen, I. I., & De Vet, H. C. W. (2009). Development of a methodological PubMed search filter for finding studies on measurement properties of measurement instruments. Quality of Life Research, 18(8), 1115-1123. https://doi.org/10.1007/s11136-009-9528-5

9. Schellingerhout, J. M., Verhagen, A. P., Heymans, M. W., Koes, B. W., De Vet, H. C., & Terwee, C. B. (2012). Measurement properties of disease-specific questionnaires in patients with neck pain: A systematic review. *Quality of Life Research*, *21*(4), 659-670. https://doi.org/10.1007/s11136-011-9965-9

10. Huang, H., Grant, J. A., Miller, B. S., Mirza, F. M., & Gagnier, J. J. (2015). A systematic review of the psychometric properties of patient-reported outcome instruments for use in patients with rotator cuff disease. *The American Journal of Sports Medicine*, *43*(10), 2572-2582. https://doi.org/10.1177/0363546514565096

11. Van Tulder, M., Furlan, A., Bombardier, C., & Bouter, L. (2003). Updated method guidelines for systematic reviews in the Cochrane Collaboration Back Review Group: *Spine*, *28*(12), 1290-1299. https://doi.org/10.1097/01.BRS.0000065484.95996.AF

12._Yi, L. C., Staboli, I. M., Kamonseki, D. H., Budiman-Mak, E., & Arie, E. K. (2015). Translation and cross-cultural adaptation of FFI to Brazilian Portuguese version: FFI – Brazil. *Revista Brasileira de Reumatologia (English Edition)*, *55*(5), 398-405. https://doi.org/10.1016/j.rbre.2014.11.005

13. Kitaoka, H. B., Alexander, I. J., Adelaar, R. S., Nunley, J. A., Myerson, M. S., & Sanders, M. (1994). Clinical rating systems for the ankle-hindfoot, midfoot, hallux, and lesser toes. *Foot & Ankle International*, *15*(7), 349–353. https://doi.org/10.1177/107110079401500701

14. Garrow, A. P., Papageorgiou, A. C., Silman, A. J., Thomas, E., Jayson, M. I. V., & Macfarlane, G. J. (2000). Development and validation of a questionnaire to assess disabling foot pain. *Pain*, *85*(1), 107-113. https://doi.org/10.1016/S0304-3959(99)00263-8

15. Richter, M., Zech, S., Geerling, J., Frink, M., Knobloch, K., & Krettek, C. (2006). A new foot and ankle outcome score: Questionnaire based, subjective, Visual-Analogue-Scale, validated and computerized. *Foot and Ankle Surgery*, *12*(4), 191-199. https://doi.org/10.1016/j.fas.2006.04.001

16. Martin, R. L., Burdett, R. G., & Irrgang, J. J. (1999). Development of the Foot and Ankle Disability Index (FADI). *The Journal of Orthopaedic & Sports Physical Therapy*, *29*, A32–A33.

17. Budiman-Mak, E., Conrad, K. J., & Roach, K. E. (1991). The Foot Function Index: A measure of foot pain and disability. *Journal of Clinical Epidemiology*, *44*(6), 561-570. https://doi.org/10.1016/0895-4356(91)90220-4

18. Bennett, P. J., Patterson, C., Wearing, S., & Baglioni, T. (1998). Development and validation of a questionnaire designed to measure foot-health status. *Journal of the American Podiatric Medical Association*, *88*(9), 419–428. https://doi.org/10.7547/87507315-88-9-419

19. Budiman-Mak, E., Conrad, K., Stuck, R., & Matters, M. (2006). Theoretical model and Rasch analysis to develop a Revised Foot Function Index. *Foot & Ankle International*, *27*(7), 519-527. https://doi.org/10.1177/107110070602700707

20. André, M., Hagelberg, S., & Stenström, C. H. (2004). The Juvenile Arthritis Foot Disability Index: Development and evaluation of measurement properties. *The Journal of Rheumatology*, *31*(12), 2488-2493. https://www.jrheum.org/content/31/12/2488

21. Robinson, J. M., Cook, J. L., Purdam, C., Visentini, P. J., Ross, J., Maffulli, N., Taunton, J. E., & Khan, K. M. (2001). The VISA-A questionnaire: A valid and reliable index of the clinical severity of Achilles tendinopathy. *British Journal of Sports Medicine*, *35*(5), 335-341. https://doi.org/10.1136/bjsm.35.5.335

22. Roos, E. M., Brandsson, S., & Karlsson, J. (2001). Validation of the Foot and Ankle Outcome Score for ankle ligament reconstruction. *Foot & Ankle International*, *22*(10), 788-794. https://doi.org/10.1177/107110070102201004

23. Munteanu, S. E., Scott, L. A., Bonanno, D. R., Landorf, K. B., Pizzari, T., Cook, J. L., & Menz, H. B. (2015). Effectiveness of customised foot orthoses for Achilles tendinopathy: A randomised controlled trial. *British Journal of Sports Medicine*, *49*(15), 989-994. https://doi.org/10.1136/bjsports-2014-093845

24. Angthong, C., Chernchujit, B., Suntharapa, T., & Harnroongroj, T. (2011). Visual Analogue Scale Foot and Ankle: Validity and reliability of Thai version of the new outcome score in subjective form. *Journal of the Medical Association of Thailand*, *94*(8), 952-957. https://www.researchgate.net/publication/51592225_Visual_analogue_scale_footandankle_Validity_and_reliability_of_Thai_version_of_the_new_outcome_score_in_subjective_form

25. Cervera-Garvi, P., Galan-Hurtado, M. H., Marchena-Rodriguez, A., Chicharro-Luna, E., Guerra-Marmolejo, C., Diaz-Miguel, S., & Ortega-Avila, A. B. (2024). Transcultural adaptation and validation of the Spanish version of the Visual Analogue Scale for the Foot and Ankle (VASFA). *Journal of Clinical Medicine*, *13*(1), 213. https://doi.org/10.3390/jcm13010213

26. Gijon-Nogueron, G., Ndosi, M., Luque-Suarez, A., Alcacer-Pitarch, B., Munuera, P. V., Garrow, A., & Redmond, A. C. (2014). Cross-cultural adaptation and validation of the Manchester Foot Pain and

Disability Index into Spanish. *Quality of Life Research*, *23*(2), 571-579. https://doi.org/10.1007/s11136-013-0507-5

27. Alshammari, S., Alshwieer, M. A. M., Dammas, S. S., Alrasheed, A. M., Alasmari, M. A., Alahmari, M. M. A., & Alazmi, A. K. (2023). Arabic translation, cross cultural adaptation, and validation of Foot Health Status Questionnaire among Saudi individuals with plantar fasciitis. *Journal of Orthopaedic Surgery and Research*, *18*, 754. https://doi.org/10.1186/s13018-023-04202-9

28. Cuesta-Vargas, A., Bennett, P., Jimenez-Cebrian, A. M., & Labajos-Manzanares, M. T. (2013). The psychometric properties of the Spanish version of the Foot Health Status Questionnaire. *Quality of Life Research*, *22*(7), 1739-1743. https://doi.org/10.1007/s11136-012-0287-3

29. Bahari, M., Hadadi, M., Vosoughi, A. R., Kordi Yoosefinejad, A., & Sobhani, S. (2022). Cross-cultural adaptation, reliability and validity of the Persian version of the Victorian Institute of Sport Assessment-Achilles questionnaire (VISA-A). *Disability and Rehabilitation*, *44*(6), 983-991. https://doi.org/10.1080/09638288.2020.1781268

30. Chang, R., Tsang, R. C.-C., Jiang, D., Liu, D., Ruan, B., Lin, G., Liu, C., & Gao, Q. (2021). Cross-cultural adaptation and measurement properties of the VISA-A questionnaire for Chinese patients with Achilles tendinopathy. *Physical Therapy in Sport*, *52*, 256-262. https://doi.org/10.1016/j.ptsp.2021.10.006

31. Amri, M. I., Alzhrani, M. M., Alanazi, A. D., Alqahtani, M. M., & Kashoo, F. Z. (2022). Cross-cultural adaptation and validation of the Arabic version of the Foot Function Index in patients with chronic lateral ankle instability. Journal of Foot and Ankle Research, 15(1), 21. https://doi.org/10.1186/s13047-022-00527-6

32. Bovonsunthonchai, S., Thong-On, S., Vachalathiti, R., Intiravoranont, W., Suwannarat, S., & Smith, R. (2020). Thai version of the Foot Function Index: A cross-cultural adaptation with reliability and validity evaluation. BMC Sports Science, Medicine and Rehabilitation, 12, 56. https://doi.org/10.1186/s13102-020-00206-8

33. Kuyvenhoven, M. M., Gorter, K. J., Zuithoff, P., Budiman-Mak, E., Conrad, K. J., & Post, M. W. M. (2002). The Foot Function Index with verbal rating scales (FFI-5pt): A clinimetric evaluationand comparison with the original FFI. *The Journal of Rheumatology*, *29*(5), 1023-1028. https://www.jrheum.org/content/29/5/1023.long

34. Sidiq, M., Chahal, A., Sharma, J., Rai, R. H., Kashoo, F. Z., Jayavelu, J., Kashyap, N., Vajrala, K. R., Veeragoudhaman, T. S., Arasu, V., & Janakiraman, B. (2024). Cross cultural adaptation and validation of

the Hindi version of Foot Function Index. Chiropractic & Manual Therapies, 32, 38. https://doi.org/10.1186/s12998-024-00563-y

35. Hernández-Sánchez, S., Poveda-Pagán, E. J., Alakhdar-Mohmara, Y., Hidalgo, M. D., Fernández-De-Las-Peñas, C., & Arias-Buría, J. L. (2018). Cross-cultural adaptation of the Victorian Institute of Sport Assessment-Achilles (VISA-A) Questionnaire for Spanish athletes with Achilles tendinopathy. Journal of Orthopaedic & Sports Physical Therapy, 48(2), 111-120. https://doi.org/10.2519/jospt.2018.7402

36. Jabbar, S., Akram, S., Khan, A. K., Arslan, S. A., Noreen, A., & Irum, S. (2024). Translation, cultural adaptation and psychometric analysis of Urdu version of Victorian; Institute of Sports Assessment: Achilles questionnaire for athletes with Achilles tendinopathy. Journal of the Pakistan Medical Association, 74(5), 897-902. https://doi.org/10.47391/JPMA.9356

37. Silbernagel, K. G., Thomeé, R., & Karlsson, J. (2005). Cross-cultural adaptation of the VISA-A questionnaire, an index of clinical severity for patients with Achilles tendinopathy, with reliability, validity and structure evaluations. BMC Musculoskeletal Disorders, 6, 12. https://doi.org/10.1186/1471-2474-6-12

38. Mørk, M., Hoksrud, A. F., Soberg, H. L., Zucknick, M., Heide, M., Groven, K. S., & Røe, C. (2022). Psychometric properties of the Norwegian Foot Function Index Revised short form‖. BMC Musculoskeletal Disorders, 23, 416. https://doi.org/10.1186/s12891-022-05374-x

39. Erichsen, J., Froberg, L., Viberg, B., Damborg, F., & Jensen, C. (2020). Danish language version of the American Orthopedic Foot and Ankle Society Ankle-Hindfoot Scale (AOFAS-AHS) in patients with ankle-related fractures. The Journal of Foot & Ankle Surgery, 59(4), 657-663. https://doi.org/10.1053/j.jfas.2019.08.027

40. Akulaev, A. A., Ivanov, A. A., Ionova, T. I., Efremov, S. M., Nikitina, T. P., Tishchenkov, K. A., & Povaliy, A. A. (2023). Linguistic and cultural adaptation of self-reported outcome questionnaires in foot and ankle-related pathologic conditions, FADI, FAAM and SEFAS, and their testing in Russian patients with different orthopedic pathology. Genij Ortopedii, 29(3), 253-264. https://doi.org/10.18019/1028-4427-2023-29-3-253-264

41. De Mesquita, G. N., De Oliveira, M. N. M., Matoso, A. E. R., De Moura Filho, A. G., & De Oliveira, R. R. (2018). Cross-cultural adaptation and measurement properties of the Brazilian Portuguese version of the Victorian Institute of Sport Assessment-Achilles (VISA-A) Questionnaire. Journal of Orthopaedic & Sports Physical Therapy, 48(7), 567-573. https://doi.org/10.2519/jospt.2018.7897

42. Alfaro-Santafé, J., Gómez-Bernal, A., Lanuza-Cerzócimo, C., Alfaro-Santafé, J.-V., Pérez-Morcillo, A., & Almenar-Arasanz, A.-J. (2021). Effectiveness of custom-made foot orthoses vs. heel-lifts in children with calcaneal apophysitis (Sever's disease): A CONSORT-compliant randomized trial. Children, 8(11), 963. https://doi.org/10.3390/children8110963

43. Andreasen, J., Mølgaard, C. M., Christensen, M., Kaalund, S., Lundbye-Christensen, S., Simonsen, O., & Voigt, M. (2013). Exercise therapy and custom-made insoles are effective in patients with excessive pronation and chronic foot pain-A randomized controlled trial. The Foot, 23(1), 22-28. https://doi.org/10.1016/j.foot.2012.12.001

44. Asgaonkar, B., & Kadam, P. (2012). Effectiveness of valgus insole on pain, gait parameters and physiological cost index of walking in flat feet in 5-15 years. *Indian Journal of Physiotherapy & Occupational Therapy*, *6*(2), 85-89.

45. Bari, A. Z., Ahmed, N., Farhan, M., Al-Shenqiti, A., & Zafar, M. S. (2025). Comparing prefabricated and 3D printed foot orthoses for the management of flat foot condition: A randomized controlled trial. American Journal of Physical Medicine & Rehabilitation, 104(4), 298-304. https://doi.org/10.1097/PHM.0000000000002585

46. Bishop, C., Thewlis, D., & Hillier, S. (2018). Custom foot orthoses improve first-step pain in individuals with unilateral plantar fasciopathy: A pragmatic randomised controlled trial. *BMC Musculoskeletal Disorders*, *19*(1), 222. https://doi.org/10.1186/s12891-018-2131-6

47. Burns, J., Wegener, C., Begg, L., Vicaretti, M., & Fletcher, J. (2009). Randomized trial of custom orthoses and footwear on foot pain and plantar pressure in diabetic peripheral arterial disease. *Diabetic Medicine : A Journal of the British Diabetic Association*, *26*(9), 893-899. https://doi.org/10.1111/j.1464-5491.2009.02799.x

48. Çağlar Okur, S., & Aydın, A. (2019). Comparison of extracorporeal shock wave therapy with custom foot orthotics in plantar fasciitis treatment: A prospective randomized one-year follow-up study. *Journal of Musculoskeletal and Neuronal Interactions*, *19*(2), 178-186. https://www.researchgate.net/publication/333756493_Comparison_of_extracorporealshock_wave_therapy_with_custom_foot_orthotics_in_plantar_fasciitis_treatment_A_prospective_randomized_one-year_follow-up_study

49. Campos, G. C. de, Rezende, M. U., Pasqualin, T., Frucchi, R., & Bolliger Neto, R. (2015). Lateral wedge insole for knee osteoarthritis: Randomized clinical trial. *São Paulo Medical Journal, 133*(1), 13-19. https://doi.org/10.1590/1516-3180.2013.6750002

50. Casado-Hernández, I., Becerro-de-Bengoa-Vallejo, R., López-López, D., Gómez-Bernal, A., & Losa-Iglesias, M. E. (2018). Aluminum foot insoles reduce plantar forefoot pressure and increase foot comfort for motorcyclists. *Prosthetics and Orthotics International*, *42*(6), 606-611. https://doi.org/10.1177/0309364618775443

51. Castro-Méndez, A., Munuera, P. V., & Albornoz-Cabello, M. (2012). The short-term effect of custom-made foot orthoses in subjects with excessive foot pronation and lower back pain: A randomized, double-blinded, clinical trial. *Prosthetics and Orthotics International*, *37*(5), 384-390. https://doi.org/10.1177/0309364612471370

52. Castro-Méndez, A., Palomo-Toucedo, I. C., Pabón-Carrasco, M., Ramos-Ortega, J., Díaz-Mancha, J. A., & Fernández-Seguín, L. M. (2021). Custom-made foot orthoses as non-specific chronic low back pain and pronated foot treatment. *International Journal of Environmental Research and Public Health*, *18*(13), 6816. https://doi.org/10.3390/ijerph18136816

53. Cho, N. S., Hwang, J. H., Chang, H. J., Koh, E. M., & Park, H. S. (2009). Randomized controlled trial for clinical effects of varying types of insoles combined with specialized shoes in patients with rheumatoid arthritis of the foot. *Clinical Rehabilitation*, *23*(6), 512-521. https://doi.org/10.1177/0269215508101737

54. Coda, A., Fowlie, P. W., Davidson, J. E., Walsh, J., Carline, T., & Santos, D. (2014). Foot orthoses in children with juvenile idiopathic arthritis: A randomised controlled trial. *Archives of Disease in Childhood*, *99*(7), 649-651. https://doi.org/10.1136/archdischild-2013-305166

55. Collins, N., Crossley, K., Beller, E., Darnell, R., McPoil, T., & Vicenzino, B. (2008). Foot orthoses and physiotherapy in the treatment of patellofemoral pain syndrome: Randomised clinical trial. *BMJ*, *337*, a1735. https://doi.org/10.1136/bmj.a1735

56. De Oliveira, H. A. V., Natour, J., Vassalli, M., Rosenfeld, A., Jennings, F., & Jones, A. (2019). Effectiveness of customized insoles in patients with Morton‘s neuroma: A randomized, controlled, double-blind clinical trial. *Clinical Rehabilitation*, *33*(12), 1898-1907. https://doi.org/10.1177/0269215519873949

57. Fellas, A., Singh-Grewal, D., Chaitow, J., Santos, D., Clapham, M., & Coda, A. (2022). Effect of preformed foot orthoses in reducing pain in children with juvenile idiopathic arthritis: A multicentre randomized clinical trial. *Rheumatology*, *61*(6), 2572-2582. https://doi.org/10.1093/rheumatology/keab765

58. Ferreira, V., Machado, L., Vilaça, A., Xará-Leite, F., & Roriz, P. (2021). Effects of tailored lateral wedge insoles on medial knee osteoarthritis based on biomechanical analysis: 12-week randomized controlled trial. *Clinical Rehabilitation*, *35*(9), 1235-1246. https://doi.org/10.1177/0269215521997988

59. Greitemann, B., Niemeyer, C., Sprekelmeyer, T., Eger, T., & Ullrich, M. (2012). „Wirken Einlagen bei der Metatarsalgie? — Eine randomisierte Kontrollgruppenstudie [Do insoles affect metatarsalgia? – A randomized controlled study]. *Fuß & Sprunggelenk*, *10*(4), 257-264. https://doi.org/10.1016/j.fuspru.2012.08.002

60. Hellstrand Tang, U., Zügner, R., Lisovskaja, V., Karlsson, J., Hagberg, K., & Tranberg, R. (2014). Comparison of plantar pressure in three types of insole given to patients with diabetes at risk of developing foot ulcers—A two-year, randomized trial. *Journal of Clinical & Translational Endocrinology*, *1*(4), 121-132. https://doi.org/10.1016/j.jcte.2014.06.002

61. Jones, R. K., Nester, C. J., Richards, J. D., Kim, W. Y., Johnson, D. S., Jari, S., Laxton, P., & Tyson, S. F. (2013). A comparison of the biomechanical effects of valgus knee braces and lateral wedged insoles in patients with knee osteoarthritis. *Gait & Posture*, *37*(3), 368-372. https://doi.org/10.1016/j.gaitpost.2012.08.002

62. Kelly, A., & Winson, I. (1998). Use of ready-made insoles in the treatment of lesser metatarsalgia: A prospective randomized controlled trial. *Foot & Ankle International*, *19*(4), 217-220. https://doi.org/10.1177/107110079801900405

63. Lewinson, R. T., Wiley, J. P., Humble, R. N., Worobets, J. T., & Stefanyshyn, D. J. (2015). Altering knee abduction angular impulse using wedged insoles for treatment of patellofemoral pain in runners: A six-week randomized controlled trial. *PLoS ONE*, *10*(7), e0134461. https://doi.org/10.1371/journal.pone.0134461

64. McRitchie, M., & Curran, M. J. (2007). A randomised control trial for evaluating over-the-counter golf orthoses in alleviating pain in amateur golfers. *The Foot*, *17*(2), 57-64. https://doi.org/10.1016/j.foot.2006.08.004

65. Menz, H. B., Auhl, M., Tan, J. M., Levinger, P., Roddy, E., & Munteanu, S. E. (2017). Predictors of response to prefabricated foot orthoses or rocker-sole footwear in individuals with first metatarsophalangeal joint osteoarthritis. *BMC Musculoskeletal Disorders*, *18*, 185. https://doi.org/10.1186/s12891-017-1558-5

66. Mills, K., Blanch, P., Dev, P., Martin, M., & Vicenzino, B. (2012). A randomised control trial of short term efficacy of in-shoe foot orthoses compared with a wait and see policy for anterior knee pain and the

role of foot mobility. *British Journal of Sports Medicine*, *46*(4), 247-252. https://doi.org/10.1136/bjsports-2011-090204

67. Moreira, E., Jones, A., Oliveira, H., Jennings, F., Fernandes, A., & Natour, J. (2016). Effectiveness of insole use in rheumatoid feet: A randomized controlled trial. *Scandinavian Journal of Rheumatology*, *45*(5), 363-370. https://doi.org/10.3109/03009742.2015.1110198

68. O'Sullivan, I. C., Crossley, K. M., Kamper, S. J., van Middelkoop, M., Vicenzino, B., Franettovich Smith, M. M., Menz, H. B., Smith, A. J., Tucker, K., O'Leary, K. T., Costa, N., & Collins, N. J. (2021). HAPPi Kneecaps! A double-blind, randomised, parallel group superiority trial investigating the effects of shoe inserts for adolescents with patellofemoral pain: Phase II feasibility study. *Journal of Foot and Ankle Research*, *14*(1), 64. https://doi.org/10.1186/s13047-021-00498-0

69. Oliveira, H. A. V., Jones, A., Moreira, E., Jennings, F., & Natour, J. (2015). Effectiveness of total contact insoles in patients with plantar fasciitis. *The Journal of Rheumatology*, *42*(5), 870-878. https://doi.org/10.3899/jrheum.140429

70. Parashar, U., & Kumar, Y. (2025). Effects of custom molded foot orthoses on balance, gait speed, and pain in postmenopausal osteoporotic women: A prospective experimental study. *Journal of Prosthetics and Orthotics*, *37*(1), 22-27. https://doi.org/10.1097/JPO.0000000000000507

71. Rannisto, S., Okuloff, A., Uitti, J., Paananen, M., Rannisto, P.-H., Malmivaara, A., & Karppinen, J. (2019). Correction of leg-length discrepancy among meat cutters with low back pain: A randomized controlled trial. *BMC Musculoskeletal Disorders*, *20*, 105. https://doi.org/10.1186/s12891-019-2478-3

72. Robert-Lachaine, X., Dessery, Y., Belzile, ÉL., & Corbeil, P. (2024). Knee braces and foot orthoses multimodal 3-month treatment of medial knee osteoarthritis in a randomised crossover trial. *Knee Surgery, Sports Traumatology, Arthroscopy*, *32*(11), 2919-2930. https://doi.org/10.1002/ksa.12312

73. Segal, N. A., Foster, N. A., Dhamani, S., Ohashi, K., & Yack, H. J. (2009). Effects of concurrent use of an ankle support with a laterally wedged insole for medial knee osteoarthritis. *PM&R*, *1*(3), 214-222. https://doi.org/10.1016/j.pmrj.2008.09.005

74. Shim, D. W., Sung, S.-Y., Chung, W.-Y., Kang, K.-Y., Park, S.-J., Lee, J. W., & Chae, D. S. (2021). Superior pedal function recovery of newly designed three spike insole over total contact insole in refractory plantar fasciitis: A randomized, double-blinded, non-inferiority study. *PLoS ONE*, *16*(7), e0255064. https://doi.org/10.1371/journal.pone.0255064

75. Simon, S., Heine, A., Dully, J., Dindorf, C., Ludwig, O., Fröhlich, M., & Becker, S. (2025). Pain reduction in patellofemoral knee patients during 3-month intervention with biomechanical and sensorimotor foot orthoses: A randomized controlled clinical study. *Biomedicines*, *13*(1), 38. https://doi.org/10.3390/biomedicines13010038

76. Tan, J. M., Menz, H. B., Crossley, K. M., Munteanu, S. E., Hart, H. F., Middleton, K. J., Smith, A. J., & Collins, N. J. (2019). The efficacy of foot orthoses in individuals with patellofemoral osteoarthritis: A randomised feasibility trial. *Pilot and Feasibility Studies*, *5*, 90. https://doi.org/10.1186/s40814-019-0469-7

77. Taseh, A., Mathur, V., Weaver, B., Hashmi, M., Vrolyk, M. A., Skolnik, J., Ashkani-Esfahani, S., & Waryasz, G. (2024). Role of insole material in treatment of plantar fasciitis: A randomized clinical trial. *Foot and Ankle Surgery*, *30*(6), 524-528. https://doi.org/10.1016/j.fas.2024.04.006

78. Toda, Y., & Tsukimura, N. (2004). A six-month followup of a randomized trial comparing the efficacy of a lateral-wedge insole with subtalar strapping and an in-shoe lateral-wedge insole in patients with varus deformity osteoarthritis of the knee. *Arthritis and Rheumatism*, *50*(10), 3129-3136. https://doi.org/10.1002/art.20569

79. Torkki, M., Malmivaara, A., Seitsalo, S., Hoikka, V., Laippala, P., & Paavolainen, P. (2001). Surgery vs orthosis vs watchful waiting for hallux valgus: A randomized controlled trial. *JAMA*, *285*(19), 2474-2480. https://doi.org/10.1001/jama.285.19.2474

80. Van Raaij, T. M., Reijman, M., Brouwer, R. W., Bierma-Zeinstra, S. M. A., & Verhaar, J. A. N. (2010). Medial knee osteoarthritis treated by insoles or braces: A randomized trial. *Clinical Orthopaedics and Related Research*, *468*(7), 1926-1932. https://doi.org/10.1007/s11999-010-1274-z

81. Vicenzino, B., Collins, N., Crossley, K., Beller, E., Darnell, R., & McPoil, T. (2008). Foot orthoses and physiotherapy in the treatment of patellofemoral pain syndrome: A randomised clinical trial. *BMC Musculoskeletal Disorders*, *9*, 27. https://doi.org/10.1186/1471-2474-9-27

82. Walther, M., Kratschmer, B., Verschl, J., Volkering, C., Altenberger, S., Kriegelstein, S., & Hilgers, M. (2013). Effect of different orthotic concepts as first line treatment of plantar fasciitis. *Foot and Ankle Surgery*, *19*(2), 103-107. https://doi.org/10.1016/j.fas.2012.12.008

83. Wegener, C., Wegener, K., Smith, R., Schott, K.-H., & Burns, J. (2016). Biomechanical effects of sensorimotor orthoses in adults with Charcot–Marie–Tooth disease. *Prosthetics and Orthotics International*, *40*(4), 436-446. https://doi.org/10.1177/0309364615579318

84. Whittaker, G. A., Munteanu, S. E., Menz, H. B., Gerrard, J. M., Elzarka, A., & Landorf, K. B. (2019). Effectiveness of foot orthoses versus corticosteroid injection for plantar heel pain: The SoOTHE randomized clinical trial. *Journal of Orthopaedic & Sports Physical Therapy*, *49*(7), 491-500. https://doi.org/10.2519/jospt.2019.8807

85. Wrobel, J. S., Fleischer, A. E., Crews, R. T., Jarrett, B., & Najafi, B. (2015). A randomized controlled trial of custom foot orthoses for the treatment of plantar heel pain. *Journal of the American Podiatric Medical Association*, *105*(4), 281-294. https://doi.org/10.7547/13-122.1

86. Wyndow, N., Crossley, K. M., Vicenzino, B., Tucker, K., & Collins, N. J. (2021). Foot orthoses and footwear for the management of patellofemoral osteoarthritis: A pilot randomized trial. *Arthritis Care and Research*, *73*(2), 240-249. https://doi.org/10.1002/acr.24098

87. Yildiz, S., Sumer, E., Zengin, H. Y., & Bek, N. (2022). Intensive physiotherapy versus home-based exercise and custom-made orthotic insoles in patients with plantar fasciitis: Pilot study. *The Foot*, *51*, 101906. https://doi.org/10.1016/j.foot.2022.101906

88. Yucel, U., Kucuksen, S., Cingoz, H. T., Anlıacik, E., Ozbek, O., Salli, A., & Ugurlu, H. (2013). Full-length silicone insoles versus ultrasound-guided corticosteroid injection in the management of plantar fasciitis: A randomized clinical trial. *Prosthetics and Orthotics International*, *37*(6), 471-476. https://doi.org/10.1177/0309364613478328

89. Yurt, Y., Şener, G., & Yakut, Y. (2019). The effect of different foot orthoses on pain and health related quality of life in painful flexible flat foot: A randomized controlled trial. *European Journal of Physical and Rehabilitation Medicine*, *55*(1), 95-102. https://doi.org/10.23736/S1973-9087.18.05108-0

90. Baker, K., Goggins, J., Xie, H., Szumowski, K., LaValley, M., Hunter, D. J., & Felson, D. T. (2007). A randomized crossover trial of a wedged insole for treatment of knee osteoarthritis. *Arthritis and Rheumatism*, *56*(4), 1198-1203. https://doi.org/10.1002/art.22516

91. Barati, K., Ebrahimi Takamjani, I., Shamsoddini, A., & Ejraei Dolatabad, H. (2022). A comparison of the biomechanical and clinical effects of a biaxial ankle-foot orthosis and lateral wedge insole in individuals with medial knee osteoarthritis. *Disability and Rehabilitation*, *44*(26), 8501-8508. https://doi.org/10.1080/09638288.2021.2019841

92. Barrios, J. A., Crenshaw, J. R., Royer, T. D., & Davis, I. S. (2009). Walking shoes and laterally wedged orthoses in the clinical management of medial tibiofemoral osteoarthritis: A one-year prospective controlled trial. *The Knee*, *16*(2), 136-142. https://doi.org/10.1016/j.knee.2008.10.002

93. Bennell, K. L., Bowles, K.-A., Payne, C., Cicuttini, F., Williamson, E., Forbes, A., Hanna, F., Davies-Tuck, M., Harris, A., & Hinman, R. S. (2011). Lateral wedge insoles for medial knee osteoarthritis: 12 month randomised controlled trial. *BMJ*, *342*, d2912. https://doi.org/10.1136/bmj.d2912

94. Maillefert, J. F., Hudry, C., Baron, G., Kieffert, P., Bourgeois, P., Lechevalier, D., Coutaux, A., & Dougados, M. (2001). Laterally elevated wedged insoles in the treatment of medial knee osteoarthritis: A prospective randomized controlled study. *Osteoarthritis and Cartilage*, *9*(8), 738-745. https://doi.org/10.1053/joca.2001.0470

95. Rafiaee, M., & Karimi, M. T. (2012). The effects of various kinds of lateral wedge insoles on performance of individuals with knee joint osteoarthritis. *International Journal of Preventive Medicine*, *3*(10), 693-698.

96. Baldassin, V., Gomes, C. R., & Beraldo, P. S. (2009). Effectiveness of prefabricated and customized foot orthoses made from low-cost foam for noncomplicated plantar fasciitis: A randomized controlled trial. *Archives of Physical Medicine and Rehabilitation*, *90*(4), 701-706. https://doi.org/10.1016/j.apmr.2008.11.002

97. Gatt, A., Formosa, C., & Otter, S. (2016). Foot orthoses in the management of chronic subtalar and talo crural joint pain in rheumatoid arthritis. *The Foot*, *27*, 27-31. https://doi.org/10.1016/j.foot.2016.03.004

98. Maddali Bongi, S., Del Rosso, A., Mikhaylova, S., Landi, G., Ferretti, B., Cavigli, E., Baccini, M., & Matucci-Cerinic, M. (2014). A comparison of two podiatric protocols for metatarsalgia in patients with rheumatoid arthritis and osteoarthritis. *Clinical and Experimental Rheumatology*, *32*(6), 855-863. https://www.clinexprheumatol.org/abstract.asp?a=7962

99. Palomo-Toucedo, I. C., Domínguez-Maldonado, G., Reina-Bueno, M., Vázquez-Bautista, M. D. C., Castillo-López, J. M., Ramos-Ortega, J., & Munuera-Martínez, P. V. (2023). Effectiveness of custom-made functional foot orthoses versus flat cushioning insoles on pain in patients with systemic lupus erythematosus. *Clinical Rehabilitation*, *37*(1), 86-97. https://doi.org/10.1177/02692155221111927

100. Powell, M., Seid, M., & Szer, I. S. (2005). Efficacy of custom foot orthotics in improving pain and functional status in children with juvenile idiopathic arthritis: A randomized trial. *The Journal of Rheumatology*, *32*(5), 943-950. https://www.jrheum.org/content/32/5/943

101. Rasenberg, N., Bierma-Zeinstra, S., Fuit, L., Rathleff, M., Dieker, A., van Veldhoven, P., Bindels, P., & van Middelkoop, M. (2021). Custom insoles versus sham and GP-led usual care in patients with plantar

heel pain: Results of the STAP-study—A randomised controlled trial. *British Journal of Sports Medicine*, *55*(5), 272-278. https://doi.org/10.1136/bjsports-2019-101409

102. Rome, K., Clark, H., Gray, J., McMeekin, P., Plant, M., & Dixon, J. (2017). Clinical effectiveness and cost-effectiveness of foot orthoses for people with established rheumatoid arthritis: An exploratory clinical trial. *Scandinavian Journal of Rheumatology*, *46*(3), 187-193. https://doi.org/10.1080/03009742.2016.1196500

103. Cambron, J. A., Dexheimer, J. M., Duarte, M., & Freels, S. (2017). Shoe orthotics for the treatment of chronic low back pain: A randomized controlled trial. *Archives of Physical Medicine and Rehabilitation*, *98*(9), 1752-1762. https://doi.org/10.1016/j.apmr.2017.03.028

104. Felson, D. T., Parkes, M., Carter, S., Liu, A., Callaghan, M. J., Hodgson, R., Bowes, M., & Jones, R. K. (2019). The efficacy of a lateral wedge insole for painful medial knee osteoarthritis after prescreening: A randomized clinical trial. *Arthritis & Rheumatology*, *71*(6), 908-915. https://doi.org/10.1002/art.40808

105. Halstead, J., Chapman, G. J., Gray, J. C., Grainger, A. J., Brown, S., Wilkins, R. A., Roddy, E., Helliwell, P. S., Keenan, A.-M., & Redmond, A. C. (2016). Foot orthoses in the treatment of symptomatic midfoot osteoarthritis using clinical and biomechanical outcomes: A randomised feasibility study. *Clinical Rheumatology*, *35*(4), 987-996. https://doi.org/10.1007/s10067-015-2946-6

106. Heide, M., Røe, C., Mørk, M., Myhre, K., Brunborg, C., Brox, J. I., & Hoksrud, A. F. (2024). Is radial extracorporeal shock wave therapy (rESWT), sham-rESWT or a standardised exercise programme in combination with advice plus customised foot orthoses more effective than advice plus customised foot orthoses alone in the treatment of plantar fasciopathy? A double-blind, randomised, sham-controlled trial. *British Journal of Sports Medicine*, *58*(16), 910-918. https://doi.org/10.1136/bjsports-2024-108139

107. Hunter, J., Spratford, W., Fearon, A., & Bousie, J. A. (2023). Do posted foot orthoses alter hip biomechanics and pain during walking in women with greater trochanteric pain syndrome? *Gait & Posture*, *99*, 35-43. https://doi.org/10.1016/j.gaitpost.2022.10.014

108. Paterson, K. L., Hinman, R. S., Metcalf, B. R., McManus, F., Jones, S. E., Menz, H. B., Munteanu, S. E., & Bennell, K. L. (2022). Effect of foot orthoses vs sham insoles on first metatarsophalangeal joint osteoarthritis symptoms: A randomized controlled trial. *Osteoarthritis and Cartilage*, *30*(7), 956-964. https://doi.org/10.1016/j.joca.2022.01.014

109. Sadler, S., Spink, M., Lanting, S., & Chuter, V. (2023). A randomised controlled trial investigating the effect of foot orthoses for the treatment of chronic nonspecific low back pain. *Musculoskeletal Care*, *21*(3), 856-864. https://doi.org/10.1002/msc.1762

110. Schmitt, A. P.-L., Liebau, K.-H., Hamm, A., Mittelmeier, W., & Schulze, C. (2025). Einlagen in der Behandlung des Knick-Knick-Senkfußes: Eine prospektive, randomisierte, doppelblinde, placebokontrollierte Vergleichsstudie zu sensomotorischen und unterstützenden Einlagen [Insoles in the treatment of pes planovalgus: A prospective, randomised, double-blind, placebo-controlled comparative trial of sensomotoric and supportive insoles]. *Orthopädie*, *54*, 61-70. https://doi.org/10.1007/s00132-024-04589-1

111. Schwarze, M., Bartsch, L. P., Block, J., Alimusaj, M., Jaber, A., Schiltenwolf, M., & Wolf, S. I. (2021). A comparison between laterally wedged insoles and ankle-foot orthoses for the treatment of medial osteoarthritis of the knee: A randomized cross-over trial. *Clinical Rehabilitation*, *35*(7), 1032-1043. https://doi.org/10.1177/0269215521993636

112. Burns, J., Crosbie, J., Ouvrier, R., & Hunt, A. (2006). Effective orthotic therapy for the painful cavus foot: A randomized controlled trial. *Journal of the American Podiatric Medical Association*, *96*(3), 205-211. https://doi.org/10.7547/0960205

113. Crosbie, J., & Burns, J. (2007). Predicting outcomes in the orthotic management of painful, idiopathic pes cavus. *Clinical Journal of Sport Medicine*, *17*(5), 337-342. https://doi.org/10.1097/JSM.0b013e31814c3e9e

114. Landorf, K. B., Keenan, A.-M., & Herbert, R. D. (2006). Effectiveness of foot orthoses to treat plantar fasciitis: A randomized trial. *Archives of Internal Medicine*, *166*(12), 1305-1310. https://doi.org/10.1001/archinte.166.12.1305

115. Munteanu, S. E., Landorf, K. B., McClelland, J. A., Roddy, E., Cicuttini, F. M., Shiell, A., Auhl, M., Allan, J. J., Buldt, A. K., & Menz, H. B. (2021). Shoe-stiffening inserts for first metatarsophalangeal joint osteoarthritis: A randomised trial. *Osteoarthritis and Cartilage*, *29*(4), 480-490. https://doi.org/10.1016/j.joca.2021.02.002

116. Lewinson, R. T., Vallerand, I. A., Collins, K. H., Wiley, J. P., Lun, V. M. Y., Patel, C., Woodhouse, L. J., Reimer, R. A., Worobets, J. T., Herzog, W., & Stefanyshyn, D. J. (2016). Reduced knee adduction moments for management of knee osteoarthritis: A three month phase I/II randomized controlled trial. *Gait & Posture*, *50*, 60-68. https://doi.org/10.1016/j.gaitpost.2016.08.027

117. Botelho, M., Pais, S., Guerreiro, C., Fernández, E., & Gonzalez, M. (2022). Impact of custom-made orthopedic footwear and plantar orthoses on quality of life and functionality of patients with diabetic neuropathic foot: A randomized clinical trial. *Diabetes Epidemiology and Management*, *5*, 100040. https://doi.org/10.1016/j.deman.2021.100040

118. Sharifian, M., Taheri, A., & Karimi, M. (2018). Comparison of the Effect of prefabricated foot orthoses on pain and quality of life in women with plantar fasciitis. *Archives Of Rehabilitation*, *19*(1), 18-25. https://doi.org/10.21859/JREHAB.19.1.18

119. Hsieh, R.-L., Peng, H.-L., & Lee, W.-C. (2018). Short-term effects of customized arch support insoles on symptomatic flexible flatfoot in children. *Medicine*, *97*(20). https://doi.org/10.1097/MD.0000000000010655

120. Dammerer, D., Fischer, F., Mayr, R., Giesinger, J., El Attal, R., & Liebensteiner, M. C. (2019). Temporary postoperative treatment with compartment-unloading knee braces or wedge insoles does not improve clinical outcome after partial meniscectomy. *Knee Surgery, Sports Traumatology, Arthroscopy*, *27*(3), 814-821. https://doi.org/10.1007/s00167-018-5106-0

121. Dwarakanathan, R., Mohanty, R. K., Sahoo, S., & Prasad, S. (2022). Efficacy of unloader knee orthosis and lateral wedge insole on static balance in medial knee osteoarthritis. *Journal of Orthopaedics, Trauma and Rehabilitation*, *29*(1). https://doi.org/10.1177/22104917221095256

122. Mahmoodi, M., Arazpour, M., & Mousavi, M. E. (2024). Comparing the effects of lower limb orthoses on knee pain, function, quality of life, and knee joint alignment in people with medial knee osteoarthritis. *Journal of Rehabilitation and Assistive Technologies Engineering*, *11*. https://doi.org/10.1177/20556683241277179

123. Salam, A., Awan, W. A., Mahmood, T., Rukh, M. S., & Seffat, N. (2019). Application of lateral wedge in knee osteoarthritis for improving pain and quality of life. *Journal of the Liaquat University of Medical and Health Sciences*, *18*(2), 146-151. https://doi.org/10.22442/jlumhs.191820618

124. De Almeida, J. S., Vanderlei, F. M., Pastre, E. C., Martins, R. A. D. M., Padovani, C. R., & Filho, G. C. (2016). Comparison of two types of insoles on musculoskeletal symptoms and plantar pressure distribution in a work environment: A randomized clinical trial. *Clinical Medicine & Research*, *14*(2), 67-74. https://doi.org/10.3121/cmr.2016.1301

125. Grim, C., Kramer, R., Engelhardt, M., John, S. M., Hotfiel, T., & Hoppe, M. W. (2019). Effectiveness of manual therapy, customised foot orthoses and combined therapy in the management of plantar fasciitis—A RCT. *Sports*, *7*(6), 128. https://doi.org/10.3390/sports7060128

126. Hatton, A. L., Williams, K., Chatfield, M. D., Hurn, S., Maharaj, J. N., Gane, E. M., Cattagni, T., Dixon, J., Rome, K., Kerr, G., & Brauer, S. G. (2023). Effects of wearing textured versus smooth shoe insoles for 12 weeks on gait, foot sensation and patient-reported outcomes, in people with multiple sclerosis: A randomised controlled trial. *Brain Impairment*, *24*(2), 148-167. https://doi.org/10.1017/BrImp.2022.33

127. King, M. G., Hon, R., Roughead, E., Kemp, J. L., Pizzari, T., Wong, J., Menz, H. B., Taylor, N. F., Harms, A., McClelland, J. A., & Semciw, A. I. (2024). Prefabricated contoured foot orthoses to reduce pain and increase physical activity in people with hip osteoarthritis: A randomised feasibility trial. *Physiotherapy Research International*, *29*(4), e2118. https://doi.org/10.1002/pri.2118

128. Norouzi, E., Bagheri, M., Alafchi, B., & Tafti, N. (2023). Analyzing the effect of varus forefoot wedge in addition to arch support on self-reported pain, function, and quality of life in patients with moderate hallux valgus: A pilot study. *Journal of Prosthetics and Orthotics*, *35*(3), 198-202. https://doi.org/10.1097/JPO.0000000000000412

129. Roos, E., Engström, M., & Söderberg, B. (2006). Foot orthoses for the treatment of plantar fasciitis. *Foot & Ankle International*, *27*(8), 606-611. https://doi.org/10.1177/107110070602700807

130. Parker, D. J., Nuttall, G. H., Bray, N., Hugill, T., Martinez-Santos, A., Edwards, R. T., & Nester, C. (2019). A randomised controlled trial and cost-consequence analysis of traditional and digital foot orthoses supply chains in a National Health Service setting: Application to feet at risk of diabetic plantar ulceration. *Journal of Foot and Ankle Research*, *12*(1), 2. https://doi.org/10.1186/s13047-018-0311-0

131. Rome, K., Gray, J., Stewart, F., Hannant, S. C., Callaghan, D., & Hubble, J. (2004). Evaluating the clinical effectiveness and cost-effectiveness of foot orthoses in the treatment of plantar heel pain: A feasibility study. *Journal of the American Podiatric Medical Association*, *94*(3), 229-238. https://doi.org/10.7547/0940229

132. Rosner, A. L., Conable, K. M., & Edelmann, T. (2014). Influence of foot orthotics upon duration of effects of spinal manipulation in chronic back pain patients: A randomized clinical trial. *Journal of Manipulative and Physiological Therapeutics*, *37*(2), 124-140. https://doi.org/10.1016/j.jmpt.2013.11.003

133. Whitford, D., & Esterman, A. (2007). A randomized controlled trial of two types of in-shoe orthoses in children with flexible excess pronation of the feet. *Foot & Ankle International*, *28*(6), 715-723. https://doi.org/10.3113/FAI.2007.0715

134. González-Sánchez, M., Ruiz-Muñoz, M., Li, G. Z., & Cuesta-Vargas, A. I. (2018). Chinese cross-cultural adaptation and validation of the Foot Function Index as tool to measure patients with foot and ankle functional limitations. *Disability and Rehabilitation*, *40*(17), 2056-2061. https://doi.org/10.1080/09638288.2017.1325944

135. Huh, J.-W., Eun, I.-S., Ko, Y.-C., Park, M.-J., Hwang, K.-M., Park, S.-H., Park, T.-H., & Park, J.-H. (2016). Reliability and validity of the Korean version of the Foot Function Index. *The Journal of Foot & Ankle Surgery*, *55*(4), 759-761. https://doi.org/10.1053/j.jfas.2016.03.011

136. Jorgensen, J. E., Andreasen, J., & Rathleff, M. S. (2015). Translation and validation of the Danish Foot Function Index (FFI-DK). *Scandinavian Journal of Medicine & Science in Sports*, *25*(4), e408-e413. https://doi.org/10.1111/sms.12331

137. Khan, S., Faulkner, S., Algarni, F. S., Almalki, A., Almansour, A., & Altowaijri, A. M. (2022). Foot Function Index for Arabic-speaking patients (FFI-Ar): Translation, cross-cultural adaptation and validation study. *Journal of Orthopaedic Surgery and Research*, *17*, 212. https://doi.org/10.1186/s13018-022-03092-7

138. Martinelli, N., Scotto, G. M., Sartorelli, E., Bonifacini, C., Bianchi, A., & Malerba, F. (2014). Reliability, validity and responsiveness of the Italian version of the Foot Function Index in patients with foot and ankle diseases. *Quality of Life Research*, *23*, 277-284. https://doi.org/10.1007/s11136-013-0435-4

139. Martinez, B. R., Staboli, I. M., Kamonseki, D. H., Budiman-Mak, E., & Yi, L. C. (2016). Validity and reliability of the Foot Function Index (FFI) questionnaire Brazilian-Portuguese version. *SpringerPlus*, *5*, 1810. https://doi.org/10.1186/s40064-016-3507-4

140. Mousavian, A., Mohammadi, A., Seyed-Hosseinian, S.-H., Shahpari, O., Elahpour, N., Orooji, A., Ebrahimzadeh, M. H., & Moradi, A. (2019). Reliability and validity of the Persian version of the Foot Function Index in patients with foot disorders. *The Archives of Bone and Joint Surgery*, *7*(3), 291-296. https://doi.org/10.22038/abjs.2019.37439.1988

141. Naal, F. D., Impellizzeri, F. M., Huber, M., & Rippstein, P. F. (2008). Cross-cultural adaptation and validation of the Foot Function Index for use in German-speaking patients with foot complaints. *Foot & Ankle International*, *29*(12), 1222-1231. https://doi.org/10.3113/FAI.2008.1222

142. Paez-Moguer, J., Budiman-Mak, E., & Cuesta-Vargas, A. I. (2014). Cross-cultural adaptation and validation of the Foot Function Index to Spanish. *Foot and Ankle Surgery*, *20*(1), 34-39. https://doi.org/10.1016/j.fas.2013.09.005

143. Pourtier-Piotte, C., Pereira, B., Soubrier, M., Thomas, E., Gerbaud, L., & Coudeyre, E. (2015). French validation of the Foot Function Index (FFI). *Annals of Physical and Rehabilitation Medicine*, *58*(5), 276-282. https://doi.org/10.1016/j.rehab.2015.07.003

144. Srimakarat, P., Jaroenarpornwatana, A., Janchai, S., & Tantisiriwat, N. (2018). Reliability and validity of Foot Function Index Thai version [FFI-TH]. *Journal of the Medical Association of Thailand*, *101*(2), 253-260. http://www.jmatonline.com/view.php?id=1888

145. Vetrano, M., Vulpiani, M. C., Erroi, D., Vadalà, A., Ferretti, A., & Saraceni, V. M. (2014). Cross-cultural adaptation and reliability of the Italian version of the Foot Function Index (FFI-I) for patients with plantar fasciitis. *The Journal of Sports Medicine and Physical Fitness*, *54*(5), 636-643. https://www.researchgate.net/publication/266381233_Cross-cultural_adaptation_and_reliability_of_the_Italian_version_of_the_Foot_Function_Index_FFI-I_for_patients_with_plantar_fasciitis

146. Wu, S.-H., Liang, H.-W., & Hou, W.-H. (2008). Reliability and validity of the Taiwan Chinese version of the Foot Function Index. *Journal of the Formosan Medical Association*, *107*(2), 111-122. https://doi.org/10.1016/S0929-6646(08)60124-2

147. Yaliman, A., Sen, E. I., Eskiyurt, N., & Budiman-Mak, E. (2014). Ayak Fonksiyon İndeksi'nin Plantar Fasiitli Hastalarda Türkçe'ye Çeviri ve Adaptasyonu [Turkish translation and adaptation of Foot Function Index in patients with plantar fasciitis]. *Türkiye Fiziksel Tip ve Rehabilitasyon Dergisi*, *60*(3), 212-222. https://doi.org/10.5152/tftrd.2014.26086

148. Bidari, S., Jalali, M., Kamali, M., & Bagheripour, B. (2021). Resaerch Paper: Translation, cultural adaptation, and psychometric evaluation of the Persian version of Foot Health Status Questionnaire. *Iranian Rehabilitation Journal*, *19*(1), 59-68. https://doi.org/10.32598/irj.19.1.1056.1

149. Ferreira, A. F. B., Laurindo, I. M. M., Rodrigues, P. T., Ferraz, M. B., Kowalski, S. C., & Tanaka, C. (2008). Brazilian version of the Foot Health Status Questionnaire (FHSQ-BR): Cross-cultural adaptation

and evaluation of measurement properties. *Clinics*, *63*(5), 595-600. https://doi.org/10.1590/S1807-59322008000500005

150. Martijn, H. A., Sierevelt, I. N., Wassink, S., & Nolte, P. A. (2023). Translation and validation of Foot Health Status Questionnaire‘ in Dutch. *The Journal of Foot & Ankle Surgery*, *62*(1), 31-34. https://doi.org/10.1053/j.jfas.2022.03.007

151. Riel, H., Jensen, M. B., Olesen, J. L., & Rathleff, M. S. (2019). Translation and cultural adaptation of a Danish version of the Foot Health Status Questionnaire for individuals with plantar heel pain. *The Foot*, *38*, 61-64. https://doi.org/10.1016/j.foot.2019.01.001

152. Mørk, M., Hoksrud, A. F., Soberg, H. L., Zucknick, M., Heide, M., Groven, K. S., & Røe, C. (2022). ―Psychometric properties of the Norwegian Foot Function Index Revised short form‖. *BMC Musculoskeletal Disorders, 23, 416*. https://doi.org/10.1186/s12891-022-05374-x

153. Rutkowski, R., Gałczyńska-Rusin, M., Gizińska, M., Straburzyński-Lupa, M., Zdanowska, A., Romanowski, M. W., Romanowski, W., Budiman-Mak, E., & Straburzyńska-Lupa, A. (2017). Adaptation and validation of the Foot Function Index-Revised short form into Polish. *BioMed Research International*, *2017*. 6051698. https://doi.org/10.1155/2017/6051698

154. Stéfani, K. C., Filho, M. V. P., Oliveira, P. R., & Wun, P. Y. L. (2017). Translation, cultural adaptation and validation of the Foot Function Index—Revised (FFI-R). *Acta Ortopedica Brasileira*, *25*(5), 188-193. https://doi.org/10.1590/1413-785220172505172107

155. Yagci, G., Erel, S., & Okunakol, V. (2020). Validation of the Turkish version of the Revised Foot Function Index for patients with foot and ankle disorders. *Foot and Ankle Surgery*, *26*(6), 624-629. https://doi.org/10.1016/j.fas.2019.08.002

156. Yi, L. C., Cabral, A. C. C., Kamonseki, D. H., Budiman-Mak, E., & Vidotto, M. C. (2017). Translation and cultural adaptation of the Revised Foot Function Index for the Portuguese language: FFI-R Brazil. São Paulo Medical Journal, 135(6), 573–577. https://doi.org/10.1590/1516-3180.2017.0183030817

157. Azadinia, F., Saeedi, H., Poorpooneh, M., Moulodi, N., & Jalali, M. (2022). Translation, cultural adaptation, and psychometric evaluation of the Manchester Foot Pain and Disability Index in Persian-speaking Iranians with foot disorders. *The Journal of Foot and Ankle Surgery*, *61*(4), 867-871. https://doi.org/10.1053/j.jfas.2021.12.010

160. Erh, B. X. Y., He, H.-G., Carter, K. F., Cheung, P. P., Tan, D. S., Wang, W., & Rome, K. (2019). Validation of the Chinese Manchester foot pain and disability index (C-MFPDI) among patients with

inflammatory arthritis. *Journal of Foot and Ankle Research*, *12*, 6. https://doi.org/10.1186/s13047-019-0316-3

161. Ferrari, S., dos Santos, F. C., Guarnieri, A. P., Salvador, N., Abou Hala Correa, A. Z., Abou Hala, A. Z., Custodio, O., & Trevisani Girl, V. F. (2008). Manchester Foot Pain Associated Disability Index in the feet of elderly people-Cultural adaptation, validation, and translation into the Portuguese language. *Revista Brasileira de Reumatologia*, *48*(6), 335–341. https://www.researchgate.net/publication/262760046_Manchester_foot_pain_associated_disability_index_in_the_feet_of_elderly_people_Cultural_adaptation_validation_and_translation_into_the_Portuguese_language

162. Kaoulla, P., Frescos, N., & Menz, H. B. (2008). Development and validation of a Greek language version of the Manchester Foot Pain and Disability Index. *Health and Quality of Life Outcomes*, *6*, 39. https://doi.org/10.1186/1477-7525-6-39

163. Pedersen, C. K., Danneskiold-Samsøe, B., Garrow, A. P., Wæhrens, E. E., Bliddal, H., Christensen, R., & Bartels, E. M. (2013). Development of a Danish language version of the Manchester Foot Pain and Disability Index: Reproducibility and construct validity testing. *Pain Research and Treatment*, *2013*, 284903. https://doi.org/10.1155/2013/284903

164. Van Der Zwaard, B. C., Terwee, C. B., Roddy, E., Terluin, B., Van Der Horst, H. E., & Elders, P. J. (2014). Evaluation of the measurement properties of the Manchester Foot Pain and Disability Index. *BMC Musculoskeletal Disorders*, *15*, 276. https://doi.org/10.1186/1471-2474-15-276

165. Alshewaier, S. A., Alotaibi, R. M., Alshabanat, A. S., & Alkathiry, A. A. (2024). Cross-cultural adaptation, translation, and validation of the Victorian Institute of Sport Assessment-Achilles Questionnaire (VISA-A) for use with Arabic-speaking patients with Achilles tendinopathy. *The Orthopaedic Journal of Sports Medicine*, *12*(6), 23259671241252649. https://doi.org/10.1177/23259671241252649

166. Dogramaci, Y., Kalaci, A., Kücükkübaş, N., Inandi, T., Esen, E., & Yanat, A. N. (2011). Validation of the VISA-A questionnaire for Turkish language: The VISA-A-Tr study. *British Journal of Sports Medicine*, *45*(5), 453-455. https://doi.org/10.1136/bjsm.2009.060236

167. Iversen, J. V., Bartels, E. M., Jørgensen, J. E., Nielsen, T. G., Ginnerup, C., Lind, M. C., & Langberg, H. (2016). Danish VISA-A questionnaire with validation and reliability testing for Danish-speaking Achilles tendinopathy patients. *Scandinavian Journal of Medicine & Science in Sports*, *26*(12), 1423-1427. https://doi.org/10.1111/sms.12576

168. Iversen, J. V., Bartels, E. M., Jørgensen, J. E., Nielsen, T. G., Ginnerup, C., Lind, M. C., & Langberg, H. (2016). Danish VISA-A questionnaire with validation and reliability testing for Danish-speaking Achilles tendinopathy patients. *Scandinavian Journal of Medicine & Science in Sports*, *26*(12), 1423-1427. https://doi.org/10.1111/sms.12576

169. Kaux, J.-F., Delvaux, F., Oppong-Kyei, J., Beaudart, C., Buckinx, F., Bartsch, V., & Bruyère, O. (2016). Cross-cultural adaptation and validation of the VISA-P and VISA-A questionnaires for French-speaking patients. *Science & Sports*, *31*(2), 65-72. https://doi.org/10.1016/j.scispo.2016.01.003

170. Kaux, J.-F., Delvaux, F., Oppong-Kyei, J., Dardenne, N., Beaudart, C., Buckinx, F., Croisier, J.-L., Forthomme, B., Crielaard, J.-M., & Bruyère, O. (2016). Validity and reliability of the French translation of the VISA-A questionnaire for Achilles tendinopathy. *Disability and Rehabilitation*, *38*(26), 2593-2599. https://doi.org/10.3109/09638288.2016.1138553

171. Keller, A., Wagner, P., Izquierdo, G., Cabrolier, J., Caicedo, N., Wagner, E., & Maffulli, N. (2018). Cross-cultural adaptation and validation of the VISA-A questionnaire for Chilean Spanish-speaking patients. *Journal of Orthopaedic Surgery and Research*, *13*, 177. https://doi.org/10.1186/s13018-018-0882-2

172. Ko, V. M. C., Lau, N. N., Qiu, J. H., Fu, S.-C., Yung, P. S.-H., & Ling, S. K.-K. (2022). Cross-cultural adaptation of Chinese Victorian Institute of Sports Assessment–Achilles (VISA-A) Questionnaire for Achilles tendinopathy. *Foot & Ankle Orthopaedics*, *7*(1). https://doi.org/10.1177/24730114221081535

173. Lohrer, H., & Nauck, T. (2009). Cross-cultural adaptation and validation of the VISA-A questionnaire for German-speaking Achilles tendinopathy patients. *BMC Musculoskeletal Disorders*, *10*, 134. https://doi.org/10.1186/1471-2474-10-134

174. Maffulli, N., Longo, U. G., Testa, V., Oliva, F., Capasso, G., & Denaro, V. (2008). Italian translation of the VISA-A score for tendinopathy of the main body of the Achilles tendon. *Disability and Rehabilitation*, *30*(20-22), 1635-1639. https://doi.org/10.1080/09638280701785965

175. Sierevelt, I., van Sterkenburg, M., Tol, H., van Dalen, B., van Dijk, N., & Haverkamp, D. (2018). Dutch version of the Victorian Institute of Sports Assessment-Achilles questionnaire for Achilles tendinopathy: Reliability, validity and applicability to non-athletes. *World Journal of Orthopedics*, *9*(1), 1-6. https://doi.org/10.5312/wjo.v9.i1.1

176. Tu, X., Tu, Z., Lin, W., & Wu, Z. (2022). The Victorian Sports Assessment Institute-Achilles Tendinopathy Questionnaire (VISA-A): Chinese cross-cultural adaptation and psychometric validation. *Health and Quality of Life Outcomes*, *20*, 111. https://doi.org/10.1186/s12955-022-02025-6

177. Adhitya, I. P. G. S., Yu, W.-Y., Saraswati, P. A. S., Winaya, I. M. N., & Lin, M.-R. (2021). Validation of the Indonesian version of the Foot and Ankle Score in patients with chronic lateral ankle instability. *Journal of Foot and Ankle Research*, *14*(1), 50. https://doi.org/10.1186/s13047-021-00488-2

178. Angthong, C. (2016). Validity and reliability of Thai version of the Foot and Ankle Outcome Score in patients with arthritis of the foot and ankle. *Foot and Ankle Surgery*, *22*(4), 224-228. https://doi.org/10.1016/j.fas.2015.09.006

179. Göksel Karatepe, A., GünaydIn, R., Kaya, T., KarlIbaş, U., & Özbek, G. (2009). Validation of the Turkish version of the Foot and Ankle Outcome Score. *Rheumatology International*, *30*(2), 169-173. https://doi.org/10.1007/s00296-009-0929-0

180. Imoto, A. M., Peccin, M. S., Rodrigues, R., & Mizusaki, J. M. (2009). Translation, cultural adaptation and validation of Foot and Ankle Outcome Score (FAOS) Questionaire into Portuguese. *Acta Ortopedica Brasileira*, *17*(4), 232-235. https://doi.org/10.1590/S1413-78522009000400008

181. Larsen, P., Boe, A. M., Iyer, A. B., & Elsøe, R. (2017). Danish translation of the Foot and Ankle Outcome Score. *Danish Medical Journal*, *64*(12), A5427. https://ugeskriftet.dk/dmj/danish-translation-foot-and-ankle-outcome-score

182. Lee, K. M., Chung, C. Y., Kwon, S. S., Sung, K. H., Lee, S. Y., Won, S. H., Lee, D. J., Lee, S. C., & Park, M. S. (2013). Transcultural adaptation and testing psychometric properties of the Korean version of the Foot and Ankle Outcome Score (FAOS). *Clinical Rheumatology*, *32* (10), 1443-1450. https://doi.org/10.1007/s10067-013-2288-1

183. Ling, S. K. K., Chan, V., Ho, K., Ling, F., & Lui, T. H. (2018). Reliability and validity analysis of the open-source Chinese Foot and Ankle Outcome Score (FAOS). *The Foot*, *35*, 48-51. https://doi.org/10.1016/j.foot.2017.12.001

184. Negahban, H., Mazaheri, M., Salavati, M., Sohani, S. M., Askari, M., Fanian, H., & Parnianpour, M. (2010). Reliability and validity of the Foot and Ankle Outcome Score: A validation study from Iran. *Clinical Rheumatology*, *29*(5), 479-486. https://doi.org/10.1007/s10067-009-1344-3

185. Pellegrini, M. J., Poniachik, R., Nuñez, A., Escudero, M. I., Carcuro, G., & Cortes, A. A. (2020). Cross-cultural adaptation and validation of the Foot and Ankle Outcome Score (FAOS) into Spanish (Chile). *Foot and Ankle Surgery*, *26*(7), 790-796. https://doi.org/10.1016/j.fas.2019.10.007

186. Sierevelt, I., van Sterkenburg, M., Tol, H., van Dalen, B., van Dijk, N., & Haverkamp, D. (2018). Dutch version of the Victorian Institute of Sports Assessment-Achilles questionnaire for Achilles tendinopathy: Reliability, validity and applicability to non-athletes. *World Journal of Orthopedics*, *9*(1), 1-6. https://doi.org/10.5312/wjo.v9.i1.1

187. Tapaninaho, K., Saarinen, A. J., Ilves, O., Uimonen, M. M., Häkkinen, A. H., Sandelin, H., & Repo, J. P. (2022). Structural validity of the Foot and Ankle Outcome Score for orthopaedic pathologies with Rasch Measurement Theory. *Foot and Ankle Surgery*, *28*(2), 193-199. https://doi.org/10.1016/j.fas.2021.03.005

188. Van Bergen, C. J. A., Sierevelt, I. N., Hoogervorst, P., Waizy, H., Van Dijk, C. N., & Becher, C. (2014). Translation and validation of the German version of the Foot and Ankle Outcome Score. *Archives of Orthopaedic and Trauma Surgery*, *134*(7), 897-901. https://doi.org/10.1007/s00402-014-1994-8

189. Van Den Akker-Scheek, I., Seldentuis, A., Reininga, I. H. F., & Stevens, M. (2013). Reliability and validity of the Dutch version of the Foot and Ankle Outcome Score (FAOS). *BMC Musculoskeletal Disorders*, *14*, 183. https://doi.org/10.1186/1471-2474-14-183

190. Gur, G., Turgut, E., Dilek, B., Baltaci, G., Bek, N., & Yakut, Y. (2017). Validity and reliability of Visual Analog Scale Foot and Ankle: The Turkish version. *The Journal of Foot & Ankle Surgery*, *56*(6), 1213-1217. https://doi.org/10.1053/j.jfas.2017.06.001

191. Kovaleva, M. A., Mogelnitskiy, A. S., & Belyaev, A. F. (2023). Валидация русскоязычной версии Визуально-аналоговой шкалы для стопы и голеностопного сустава — Visual Analog Scale Foot and Ankle (VAS FA) [Validation of Russian-language version of Visual Analog Scale Foot and Ankle (VAS FA)]. *Rossijskij Osteopaticeskij Zurnal*, *3*, 34-45. https://doi.org/10.32885/2220-0975-2023-3-34-45

192. Nair, A. V., Shamsuddin, K., John, P. S., Hämäläinen, J. A., & Kurien, M. A. (2015). Correlation of Visual Analogue Scale Foot and Ankle (VAS-FA) to AOFAS score in malleolar fractures using Indian language questionnare. *Foot and Ankle Surgery*, *21*(2), 125-131. https://doi.org/10.1016/j.fas.2014.10.006

193. Repo, J. P., Tukiainen, E. J., Roine, R. P., Kautiainen, H., Lindahl, J., Ilves, O., Järvenpää, S., & Häkkinen, A. (2018). Reliability and validity of the Finnish version of the Visual Analogue Scale Foot and Ankle (VAS-FA). *Foot and Ankle Surgery*, *24*(6), 474-480. https://doi.org/10.1016/j.fas.2017.05.009

194. Zarei, H., Nosratpour, M., Moteshakereh, S. M., Mahdavi, M., & Sabaghzadeh, A. (2025). Visual Analog Scale Foot and Ankle (VAS-FA): Reliability and validity of the Persian version. *Foot and Ankle Surgery*. https://doi.org/10.1016/j.fas.2025.02.004

195. Eun, I.-S., Oh, Y. S., Kim, J., & Jang, W. (2024). Reliability and validity of the Korean version of Foot and Ankle Disability Index. *The Journal of Foot & Ankle Surgery*, *63*(1), 33-35. https://doi.org/10.1053/j.jfas.2023.08.007

196. Hale, S. A., & Hertel, J. (2005). Reliability and sensitivity of the Foot and Ankle Disability Index in subjects with chronic ankle instability. *Journal of Athletic Training*, *40*(1), 35-40.

197. Leigheb, M., Rava, E., Vaiuso, D., Samaila, E. M., Pogliacomi, F., Bosetti, M., Grassi, F. A., & Sabbatini, M. (2020). Translation, cross-cultural adaptation, reliability, and validation of the Italian version of the Foot and Ankle Disability Index (FADI). *Acta Biomedica*, *91*(4-S), 160-166. https://doi.org/10.23750/abm.v91i4-S.9544

198. Popli, A., Goyal, K., Chatterjee, S., & Goyal, M. (2024). Validity and reliability of Hindi version of Foot and Ankle Disability Index in patients with chronic recurrent lateral ankle sprain. *Foot and Ankle Surgery*, *30*(1), 27-31. https://doi.org/10.1016/j.fas.2023.08.002

199. Alhadhoud, M., Alsiri, N., Alsaffar, M., & Glazebrook, M. (2020). Cross-cultural adaptation and validation of an Arabic version of the American Orthopedics Foot and Ankle Score (AOFAS). *Foot and Ankle Surgery*, *26*(8), 876-882. https://doi.org/10.1016/j.fas.2019.11.001

200. Analay Akbaba, Y., Celik, D., & Ogut, R. T. (2016). Translation, cross-cultural adaptation, reliability, and validity of Turkish version of the American Orthopaedic Foot and Ankle Society Ankle-Hindfoot Scale. *The Journal of Foot and Ankle Surgery*, *55*(6), 1139-1142. https://doi.org/10.1053/j.jfas.2016.06.001

201. De Boer, A. S., Tjioe, R. J. C., Van Der Sijde, F., Meuffels, D. E., Den Hoed, P. T., Van Der Vlies, C. H., Tuinebreijer, W. E., Verhofstad, M. H. J., & Van Lieshout, E. M. M. (2017). The American Orthopaedic Foot and Ankle Society Ankle-Hindfoot Scale; translation and validation of the Dutch language version for ankle fractures. *BMJ Open*, *7*(8), e017040. https://doi.org/10.1136/bmjopen-2017-017040

202. Fomichev, V. A., Sorokin, E. P., Konovalchuk, N. S., Pashkova, E. A., & Sereda, A. P. (2023). Cross-cultural adaptation and validation of the Russian-language version of the American Orthopaedic Foot and Ankle Society Ankle-Hindfoot Scale (AOFAS-AHS). *Traumatology and Orthopedics of Russia*, *29*(4), 78-86. https://doi.org/10.17816/2311-2905-16494

203. Kostuj, T., Schaper, K., Baums, M. H., & Lieske, S. (2014). Eine Validierung des AOFAS-Ankle-Hindfoot-Scale für den deutschen Sprachraum [German validation of the AOFAS Ankle Hindfoot Scale]. *Fuß & Sprunggelenk*, *12*(2), 100-106. https://doi.org/10.1016/j.fuspru.2014.02.002

204. Leigheb, M., Janicka, P., Andorno, S., Marcuzzi, A., Magnani, C., & Grassi, F. (2016). Italian translation, cultural adaptation and validation of the ―American Orthopaedic Foot and Ankle Society's (AOFAS) Ankle-Hindfoot Scale‖. *Acta Biomedica*, *87*(1), 38-45. https://www.researchgate.net/publication/302910000_Italian_translation_cultural_adaptation_and_validation_of_the_American_Orthopaedic_Foot_and_Ankle_Society's_AOFAS_ankle-hindfoot_scale

205. Makhmetova, M., Baktybergen, K., Raimagambetov, Y., Balbossynov, B., & Saginova, D. (2024). Cross-cultural adaptation and validation of the Kazakh version of American Orthopaedic Foot and Ankle Society Score (AOFAS) questionnaire. *Acta Biomedica*, *95*(5), e2024177. https://doi.org/10.23750/abm.v95i5.16236

206. Rodrigues, R. C., Masiero, D., Mizusaki, J. M., Imoto, A. M., Peccin, M. S., Cohen, M., & Alloza, J. F. M. (2008). Translation, cultural adaptation and validation of the ―American Orthopaedic Foot and Ankle Society's (AOFAS) Ankle-Hindfoot Scale‖. *Acta Ortopedica Brasileira*, *16*(2), 107-111. https://doi.org/10.1590/S1413-78522008000200009

207. Seyed Hoseinian, S. H., Hassankhani, G. G., Bagheri, F., Alavi, N., Shojaie, B., & Mousavian, A. (2018). Validation of the Persian version of the American Orthopedic Foot and Ankle Society Score (AOFAS) Questionnaire. *The Archives of Bone and Joint Surgery*, *6*(3), 233-239. https://doi.org/10.22038/abjs.2018.28241.1729

208. Vosoughi, A. R., Roustaei, N., & Mahdaviazad, H. (2018). American Orthopaedic Foot and Ankle Society Ankle–Hindfoot Scale: A cross-cultural adaptation and validation study from Iran. *Foot and Ankle Surgery*, *24*(3), 219-223. https://doi.org/10.1016/j.fas.2017.02.007

209. Kalaycioglu, O., Ayanoglu, T., Yilmaz, Ö. F., & Turhan, M. T. (2023). A cross-cultural adaptation and validation of the Turkish version of American Orthopaedic Foot and Ankle Society Metatarsophalangeal-Interphalangeal Scale (AOFAS-MTP-IP) for the hallux. *Joint Diseases and Related Surgery*, *34*(2), 305-314. https://doi.org/10.52312/jdrs.2023.1141

210. Leigheb, M., Vaiuso, D., Rava, E., Pogliacomi, F., Samaila, E. M., Grassi, F. A., & Sabbatini, M. (2019). Translation, cross-cultural adaptation, reliability, and validation of the Italian version of the

American Orthopaedic Foot and Ankle Society-Meta- TarsoPhalangeal-InterPhalangeal Scale (AOFAS-MTP-IP) for the hallux. *Acta Biomedica*, *90*(12-S), 118-126. https://doi.org/10.23750/abm.v90i12-S.8978

211. Mahdaviazad, H., Kardeh, B., & Vosoughi, A. R. (2020). American Orthopedic Foot and Ankle Society Hallux Metatarsophalangeal-Interphalangeal Joint Scale: A cross-cultural adaptation and validation study in the Persian language. *The Journal of Foot & Ankle Surgery*, *59*(4), 729-732. https://doi.org/10.1053/j.jfas.2020.01.006

212. Molano Castro, J. D., Sardoth Álvarez, R., Franco Betancur, A., & Vargas Montenegro, G. E. (2023). Translation, cultural adaptation, and validation of the American Orthopedic Foot and Ankle Society Scale in patients with hallux valgus in Colombia. *The Journal of Foot & Ankle Surgery*, *62*(3), 511-518. https://doi.org/10.1053/j.jfas.2022.12.006

213. Ziroglu, N., Birinci, T., Koluman, A., Şahbaz, Y., Çiftçi, M. U., Baca, E., & Duramaz, A. (2023). Reliability and validity of the Turkish version of the American Orthopaedic Foot and Ankle Society Hallux Metatarsophalangeal-Interphalangeal Joint Scale. *Foot & Ankle Specialist*. https://doi.org/10.1177/19386400231214285
